# Human induced-T-to-natural killer cells have potent anti-tumour activities

**DOI:** 10.1186/s40364-022-00358-4

**Published:** 2022-03-24

**Authors:** Zhiwu Jiang, Le Qin, Yuou Tang, Rui Liao, Jingxuan Shi, Bingjia He, Shanglin Li, Diwei Zheng, Yuanbin Cui, Qiting Wu, Youguo Long, Yao Yao, Zhihui Wei, Qilan Hong, Yi Wu, Yuanbang Mai, Shixue Gou, Xiaoping Li, Robert Weinkove, Sam Norton, Wei Luo, Weineng Feng, Hongsheng Zhou, Qifa Liu, Jiekai Chen, Liangxue Lai, Xinwen Chen, Duanqing Pei, Thomas Graf, Xingguo Liu, Yangqiu Li, Pentao Liu, Zhenfeng Zhang, Peng Li

**Affiliations:** 1 China-New Zealand Joint Laboratory of Biomedine and Health, State Key Laboratory of Respiratory Disease, Guangdong Provincial Key Laboratory of Stem Cell and Regenerative Medicine, Chinese Academy of Sciences Key Laboratory of Stem Cell and Regenerative Medicine, Guangzhou Institutes of Biomedicine and Health, Chinese Academy of Sciences, Guangzhou, China; 2 Department of Radiology; Guangdong Provincial Education Department Key Laboratory of Nano-Immunoregulation Tumour Microenvironment; Guangzhou Key Laboratory for Research and Development of Nano-Biomedical Technology for Diagnosis and Therapy, the Second Affiliated Hospital of Guangzhou Medical University, Guangzhou, China; 3 Guangdong Zhaotai InVivo Biomedicine Co. Ltd., Guangzhou, China; 4 Bioland Laboratory (Guangzhou Regenerative Medicine and Health Guangdong Laboratory), Guangzhou, China; 5 Centre for Genomic Regulation, The Barcelona Institute of Science and Technology, Barcelona, Spain; 6 Zhongshan School of Medicine, Sun Yat-Sen University, Guangzhou, China; 7 Cancer Immunotherapy Programme, Malaghan Institute of Medical Research, Wellington, New Zealand; 8 Nanix Limited, Dunedin, New Zealand; 9 Clinical Research Institute, The First People’s Hospital of Foshan, Foshan, China; 10 Department of Head and Neck/Thoracic Medical Oncology, The First People’s Hospital of Foshan, Foshan, Guangdong China; 11 Department of Hematology, Nanfang Hospital, Guangzhou, China; 12 School of Life Sciences, Westlake University, Hangzhou, China; 13 Institute of Hematology, Medical College, Jinan University, Guangzhou, China; 14 School of Biomedical Sciences, Stem Cell and Regenerative Medicine Consortium, Li Ka Shing Faculty of Medicine, The University of Hong Kong, Hong Kong, SAR China; 15 Centre for Regenerative Medicine and Health, Hong Kong Institute of Science & Innovation, Chinese Academy of Sciences, Hong Kong, SAR China

**Keywords:** BCL11B, CRISPR/Cas9, T cells, Immunotherapy

## Abstract

**Background:**

Adoptive cell therapy (ACT) is a particularly promising area of cancer immunotherapy, engineered T and NK cells that express chimeric antigen receptors (CAR) are being explored for treating hematopoietic malignancies but exhibit limited clinical benefits for solid tumour patients, successful cellular immunotherapy of solid tumors demands new strategies.

**Methods:**

Inactivation of BCL11B were performed by CRISPR/Cas9 in human T cells. Immunophenotypic and transcriptional profiles of sg*BCL11B* T cells were characterized by cytometer and transcriptomics, respectively. sg*BCL11B* T cells are further engineered with chimeric antigen receptor. Anti-tumor activity of ITNK or CAR-ITNK cells were evaluated in preclinical and clinical studies.

**Results:**

We report that inactivation of BCL11B in human CD8^+^ and CD4^+^ T cells induced their reprogramming into induced T-to-natural killer cells (ITNKs). ITNKs contained a diverse TCR repertoire; downregulated T cell-associated genes such as *TCF7* and *LEF1*; and expressed high levels of NK cell lineage-associated genes. ITNKs and chimeric antigen receptor (CAR)-transduced ITNKs selectively lysed a variety of cancer cells in culture and suppressed the growth of solid tumors in xenograft models. In a preliminary clinical study, autologous administration of ITNKs in patients with advanced solid tumors was well tolerated, and tumor stabilization was seen in six out nine patients, with one partial remission.

**Conclusions:**

The novel ITNKs thus may be a promising novel cell source for cancer immunotherapy.

**Trial registration:**

ClinicalTrials.gov, NCT03882840. Registered 20 March 2019-Retrospectively registered.

**Supplementary Information:**

The online version contains supplementary material available at 10.1186/s40364-022-00358-4.

## Introduction

Adoptive cell therapy (ACT) is a particularly promising area of cancer immunotherapy [1–3]. Unlike T cells, NK cells play an important role in immune surveillance by targeting tumor cells that downregulate HLA class I molecules or express stress markers [4]. Engineered T and NK cells that express chimeric antigen receptors (CARs) or cancer-specific T cell receptor (TCR) transgenes with PD-1 ablation have been successfully used to treat hematopoietic malignancies but exhibit limited clinical benefits for solid tumor patients [3, 5–13]. New immune cell sources that recognize and eliminate solid tumor cells would therefore be desirable.

T cell lineage specification and commitment involve progenitors homing to the thymus [14, 15]. Bcl11b is a transcription factor required for T cell commitment [16–21]. Its upregulation represents a “point of no return” in T cell lineage determination [22] and depends on Notch signaling [23] and a cohort of transcription factors, such as Runx1, Tcf1, and Gata3 [24–26]. T cell progenitors in *Bcl11b* knockout mice fail to undergo β-selection [27]. Bcl11b is also required for different T cell subsets [28–31]. Bcl11b-deficient T cell progenitors preserve NK and myeloid potentials [16–18]. Acute inactivation of *Bcl11b* in mature T cells induces their reprogramming into induced T-to-NK cells (ITNKs) [16]. These cells acquire the expression of NK cell receptors (NKp46) and can recognize and kill both MHC-I-positive and MHC-I-negative/low tumor cells without attacking normal cells [16]. Mechanistically, Bcl11b directly represses the transcription of Id2, which governs NK cell fate, and Zbtb16, which is crucial in innate-type T cells and innate lymphoid cells [20, 32–34].

Human T cell development also requires BCL11B [35–37]. Patients carrying BCL11B mutations exhibit primary immunodeficiency caused by T cell deficiency [38–40]. Dysregulation of BCL11B has been implicated in T cell leukemias [41, 42]. The inhibition of BCL11B induces apoptosis in T-ALL [43–45]. In contrast, knockdown of BCL11B in normal mature human T cells does not affect viability but rather upregulates the expression of ID2 [43]. In addition, the suppression of BCL11B by chimeric antigen receptor (CAR) expression in human lymphoid progenitors represses the expression of T cell-associated genes, including *IL7R*, *GATA3*, and *NOTCH3*, and increases the expression of *ID2* and *GZMB* [46].

The roles of BCL11B in mature human T cells during homeostasis have not yet been fully elucidated. Here, we report that inactivating BCL11B in multiple human T cell subsets reprogrammed them into induced T-to-NK cells (ITNKs). ITNKs retained a functional TCR, upregulated NK cell-associated markers and transcription factors, and contained elongated tubular mitochondria. Mechanistically, BCL11B directly repressed NK-cell associated transcription factors for the maintenance of T cell identity. ITNKs recognized and efficiently lysed cancer cells in culture, in organoids and in murine cancer models. In a clinical investigation, transplanted autologous ITNKs exhibited tumoricidal activities in patients with refractory and advanced solid tumors with no severe adverse effects. The molecular, cellular, preclinical and clinical studies presented here demonstrate that human ITNKs could be further explored as a new cell source for cell-based cancer immunotherapy.

## Methods

### Reagents and antibodies

SR11302 (A8185, APExBIO) was used for the rescue experiment. All antibodies used in the study for fluorescence-activated cell sorting, flow cytometry and cellular stimulation are listed in Table S10. All sgRNAs used in the study are listed in Table S10.

### Isolation, transduction, and expansion of primary human T lymphocytes, γδ T cells and NK cells

For all preclinical experiments in this study, PBMCs were isolated from cord blood or from healthy adult donors (median age 33 years, range 29–50 years; two females and three males) using Lymphoprep (Stem Cell Technologies, Vancouver, Canada). T cells were negatively selected from PBMCs using a MACS Pan T Cell Isolation Kit (Miltenyi Biotec, Bergisch Gladbach, Germany) and activated MACS GMP T Cell TransAct (Miltenyi Biotec, Bergisch Gladbach, Germany), in 5 μl at a bead: cell ratio of 1:2 and a density of 2.5 × 10^6^ cells/ml for 1 day in T551-H3 (Takara, Japan) medium supplemented with 5% heat-inactivated fetal bovine serum (FBS), 500 U/ml recombinant human IL-2, 10 mM HEPES, 2 mM glutamine and 1% penicillin/streptomycin. γδ T cells were cultured in RPMI 1640 medium supplemented with 10% FBS, antibiotics, IL-2 (100 IU/ml), vitamin C (70 μΜ) and ZOL (50 μM, Τ6739, TargetMol) as reported by Yin et al. [47]. NK cells were cultured in T551-H3 medium with 5% FBS and 500 U/mL IL-2 at an initial density of 1 × 10^6^ cells/ml. Briefly, CB or PBMCs were directly activated with NK Cell Activation/ Expansion Kit (130–094-483, Miltenyi Biotec, Germany), which contains microbeads loaded with NKp46 and CD2 antibodies and were cultured supplemented with IL-2 (500 U/mL). Healthy PBMC donors provided informed consent for the use of their samples for research purposes, and all procedures were approved by the Research Ethics Board of the Guangzhou Institutes of Biomedicine and Health, Chinese Academy of Sciences (GIBH).

### Induction and expansion of ITNKs and CAR-ITNKs

For electroporation, on postactivation day 1, T cells from CB were electroporated with sg*BCL11B* (5 μg plasmid per 1 × 10^7^ T cells) or sgBCL11B/PiggyBac (PB)-CAR vectors (3 μg of sg*BCL11B* plasmid, 2 μg of PB-CAR plasmid, and 1 μg of PBase plasmid per 1 × 10^7^ T cells) using an Amaxa Nucleofector 2b (Amaxa® Human T Cell Nucleofector® Kit, Lonza, Germany) with electroporation program T-023. Twelve hours after electroporation, T cells were cultured in T cell culture medium containing rh-IL2 (500 U/mL). Cas9 RNPs were prepared immediately before experiments by incubating 20 μM Cas9 with 20 μM sgRNA at a 1:1 ratio in Human T Cell Nucleofector buffer at 37 °C for 10 min to a final concentration of 10 μM [9]. For lentivirus transduction, on postactivation day 1, T cells were transfected with lentivirus at an MOI of 5. Twelve hours after transduction, T cells were electroporated with sg*BCL11B* (5 μg plasmids per 1 × 10^7^ T cells). Subsequently, fresh medium was added every 2 days to maintain cell density within the range of 0.5–1 × 10^6^ cells/ml. CD3^+^NKp46^+^ T cells were defined as ITNKs and used in in vitro and in vivo experiments.

### Cell lines

HeLa/ HeLa-GL (Human cervical cancer), SK-OV-3-GL (Human ovarian cancer), and human HCC cell lines (HepG2/HepG2-GL) were maintained in Dulbecco’s modified Eagle’s medium (DMEM) (Gibco, Grand Island, NY, USA). Other cell lines, including K562, K562-GL, CD19^+^K562-GL, NALM-6, NALM-6-GL and OKT3^+^ NALM-6-GL (leukemia), were maintained in RPMI-1640 medium supplemented with 10% heat-inactivated FBS (Gibco, Grand Island, NY, USA), 10 mM HEPES, 2 mM glutamine (Gibco, Grand Island, NY, USA) and 1% penicillin/streptomycin (Gibco, Grand Island, NY, USA). All cells were cultured at 37 °C in an atmosphere of 5% carbon dioxide. Cell line identity was confirmed by STR sequencing.

### Functional assays

For plate-bound antibody stimulation assays, 96-well flat-bottom plates (MaxiSorp, Nunc, Thermo Fisher, USA) were precoated with different antibodies. A total of 1 × 10^5^ cells were added to the wells in complete medium. After 1 hour of incubation, GolgiStop (554,724, Biosciences, USA) was added, and the plates were incubated for four additional hours at 37 °C. Afterwards, the cells were washed, fixed, permeabilized and stained with anti-IFN-γ and IL-2 antibodies.

### In vitro killing assays

The K562-GL, HeLa-GL, NALM6-GL, SK-OV-3-GL and HepG2-GL target cells were incubated with the indicated killing cells at the indicated ratio in triplicate wells of U-bottomed 96-well plates. Target cell viability was monitored 18 h later by adding 100 μl of the substrate D-luciferin (potassium salt) (Cayman Chemical, Michigan, USA) at 150 μg/ml to each well. Background luminescence was negligible (< 1% of the signal from wells containing only target cells). Spheroids of PDOs were obtained as described in previous studies [48]. Briefly, 5 × 10^5^ cells were seeded into wells of a 24-well Kuraray ultralow attachment plate (round-bottom type; Elplasia, Japan) on day 0. On day 2, ITNKs, T cells, NK cells, or culture medium only (as blank control) was cocultured with spheroids in 96-well U-bottomed plates. Cytotoxicity was assayed at 72 h post coculture using a CellTiter-Glo® 3D Cell Viability Assay (G9683, Promega, USA). The percent cytotoxicity (killing %) values were calculated as (blank signal – experimental signal)/blank signal× 100%.

### Xenograft models and in vivo assessment

Animal experiments were performed in the Laboratory Animal Center of Guangzhou Institutes of Biomedicine and Health (GIBH), and all animal procedures were approved by the Animal Welfare Committee of GIBH. All protocols were approved by the relevant Institutional Animal Care and Use Committee (IACUC). NSI mice [49] were maintained in specific pathogen-free (SPF)-grade cages and were provided autoclaved food and water. Direct injection of the indicated tumor cells or leukemia cells in 200 μL of PBS was performed to establish subcutaneous (flank) or intravenous (tail vein) tumors, respectively. At the indicated time for each experiment, 2.5–5 × 10^6^ of the indicated killing cells in 200 μL of PBS were adoptively transferred to tumor-bearing mice systemically by tail vein injection. Peripheral blood was obtained by retro-orbital bleeding. The body weight of the mice was measured every 2 or 3 days as indicated. The xenografted mice were then randomized into different groups. The sample size of each group for all mouse experiments was *n* ≥ 5. Tumors were measured every 3 days with a caliper. The tumor volume was calculated using the following equation: (length×width^2^)/2. In vivo whole-body imaging of luciferase-labeled cells was performed using a cooled CCD camera system (IVIS 100 Series Imaging System, Xenogen, Alameda, CA, USA) [50]. Firefly D-luciferin (potassium salt) was injected at 75 mg/kg. Mice were imaged 5 min after the injection of the substrate. Quantification of total and average emissions was performed using Living Image software. ITNKs and control T cells were cultured under the same conditions (T551-H3 (Takara, Japan) medium supplemented with 5% heat-inactivated FBS, 500 U/ml recombinant human IL-2, 10 mM HEPES, 2 mM glutamine and 1% penicillin/streptomycin), and the expression of CD45RA and CD45RO was characterized before infusion into xenografts. To investigate the function of ITNKs upon adoptive transfer, we first examined the distribution and persistence of ITNKs in vivo. Flow cytometry was used to monitor the proportions of ITNKs in the PB, spleen, bone marrow (BM), liver and lungs on days 7, 14, 21, and 28.

### Flow cytometry and cell sorting

Flow cytometric analysis was performed on a FACSCanto or FACS Fortessa instrument (BD, USA). FACS was performed on the FACSAria II platform (BD, USA). Surface staining for flow cytometry and cell sorting was performed by pelleting cells and resuspending in 50 μl of FACS buffer (2% FBS in PBS) with antibodies for 30 min at 4 °C in the dark. For intracellular staining, cells were fixed and permeabilized with the Foxp3/Transcription Factor Staining Buffer Set (Thermo Fisher, USA), washed, blocked with mouse or rabbit serum and incubated with antibodies for 30 min at 4 °C. Cells were washed once in FACS buffer before resuspension.

### Imaging flow cytometry

Surface staining was performed as described above. Cells were washed with 1 × PBS buffer containing 0.5 mM EDTA and 0.2% BSA at pH 7.2 and suspended at a concentration of 1–2 × 10^6^/mL. Positive staining for each antibody-fluorophore combination was determined using FMO controls. Samples were acquired on an Amnis ImageStream X Mark II instrument equipped with 405 nM, 488 nM, 561 nM, and 640 nM lasers utilizing INSPIRE software (Amnis, Seattle, WA). Automatic compensation was performed with a single color, followed by manual adjustment and analysis using IDEAS 6.0 software (Amnis, Seattle, WA).

### Cytokine release assays

ITNKs, T cells and NK cells (2 × 10^5^) were washed twice and mixed with 2 × 10^5^ K562 cells in 200 mL of complete medium. Cells were incubated for the indicated time at 37 °C in 5% CO_2_ for 18 h. Then, supernatants were collected and stored at − 20 °C for further measurement. The concentrations of cytokines were quantified by a multiplex immunoassay (Milliplex MAP, Millipore, USA) using a kit that detects 21 different cytokines (Cat.# HSTCMAG28SPMX21).

### PCR genotyping

To extract genomic DNA, sorted cells were incubated in 400 μl of lysis buffer (50 mM Tris pH 8.0, 100 mM NaCl, 25 mM EDTA pH 8.0, 0.5% SDS, and 0.5 mg/ml proteinase K) at 65 °C for 2 h. Genomic DNA was precipitated by adding 500 μl of isopropanol to the cell lysis buffer. After centrifugation, the DNA was washed once with 500 μl 70% ethanol and air dried before being resuspended and used as a template for PCR. Exon 2 and exon 3 of the *BCL11B* locus were detected by PCR with the primers described below. Exon 2: Forward 5′-tcctcgaagaagacgagggt-3′, Reverse 5′-gttctcctgcttgggacaga-3′ and Exon 3: Forward 5′-tgatcacttcacctctgcgt’, R 5′- aatgcattctgggagcaaga-3′.

### Protein isolation and immunoblotting

Cells were lysed with RIPA buffer (Pierce, Rockford, Illinois, USA), and the protein concentration was quantified using the BCA Protein Assay kit (Pierce, Rockford, Illinois, USA). Samples were loaded onto an 8–12% SDS-PAGE gel, transferred to a PVDF membrane, and sequentially probed with primary antibodies. A species-matched HRP-conjugated secondary antibody was then added, and proteins were detected by autoradiography using an enhanced chemiluminescence kit (ECL Plus, General Electric Healthcare, Little Chalfont, UK).

### Histological analysis

Organ or tissue samples were fixed in 10% formalin, embedded in paraffin, sectioned at 4 μm thickness, and stained with hematoxylin and eosin or the indicated antibodies. Images were obtained on a microscope (Leica DMI6000B, Leica Microsystems, Wetzlar, Germany).

### CyTOF sample preparation and acquisition

Cells from culture suspensions were stained for viability with 5 mM cisplatin in PBS (Fluidigm, USA) for 5 min on ice and then washed with PBS with 0.5% BSA and 0.02% NaN_3_. Cells were suspended in Fc receptor blocking mix, incubated for 20 min on ice, and subsequently stained with metal-labeled monoclonal antibody (mAb) cocktails against cell surface molecules for 30 min on ice. Antibodies were either purchased preconjugated from Fluidigm or conjugated in-house using mass cytometry antibody conjugation kits (Fluidigm, USA) according to the manufacturer’s instructions. Cells were then washed and stained with 200 μl of 1:4000 ^191/193^Ir DNA intercalator (Fluidigm) diluted in Fix and Perm (Fluidigm) at 4 °C overnight. After treatment with fixation/permeabilization buffer (Thermo Fisher, USA), cells were further incubated with a metal-labeled mAb cocktail against intracellular proteins. At the time of acquisition, cells were washed once with PBS with 0.5% BSA and 0.02% NaN_3_, once with ddH_2_O, and then suspended in ddH_2_O containing bead standards (Fluidigm, USA) to approximately 1 × 10^6^ cells per ml. Samples were subsequently acquired on a CyTOF instrument (Fluidigm, USA) at an event rate of < 300 events/second [51]. Antibody information is given in Table S10.

### Bulk RNA sequencing

mRNA extracted from purified T, ITNK and NK cells was prepared according to the TruSeqTM RNA Sample Preparation Guide, and sequencing was performed. Sequenced reads were trimmed to eliminate adaptor sequences and masked to remove low complexity or low-quality sequences. The number of raw reads mapped to genes was calculated by RSEM (rsem-1.2.4), and the sample results were combined and normalized in EDAseq (1.99.1). Gene expression fold changes were calculated using normalized raw reads. The downstream analysis used glbase scripts.

### Single-cell RNA sequencing and analysis

To capture single-cell transcriptomic information related to BCL11B inactivation in T cells, we collected sg*BCL11B*-edited T cells from days 2–10 (D2, D4, D6, D8, D10) for 10x single-cell RNA-seq. We prepared libraries following the Chromium Single Cell 30 Reagent Kit User Guide. The single-cell libraries were quantified by a high sensitivity Quant-iT dsDNA Assay Kit (Thermo Fisher) on a Qubit 2.0 instrument and then sequenced on an Illumina HiSeq 2500 by Guangzhou Gene Denovo. For data analysis, cellranger-2.1.1 was used to map the 10x single-cell RNA-seq data. The read1 data of pooled cells were split into single-cell data using the barcode sequences contained in the first 16 bp. The next 10 bp were recorded as unique molecular identifiers (UMIs). Read2 75 bp sequences were aligned to the mm10 genome. We used Seurat (v2.3.0) for preprocessing of the data. We excluded cells with fewer than 2500 detected genes. Overall, 11,011 (D2), 6562 (D4), 7464 (D6), 7426 (D8) and 6148 (D10) cells met the quality control criteria and were used for further analysis [52]. Raw scRNA-sequencing data from human blood and splenic NK cells were downloaded from the database listed in Crinier et al. [53].

### TCRβ sequencing

cDNA was first generated and amplified by using a human TCRβ profiling kit (635,014, Clontech, Takara). Libraries were sequenced on a HiSeq4000 platform (Illumina, USA). The clean reads were aligned and assembled using MiXCR. The TCRβ clonotypes were exported by parameter ‘--chains’ in the export Clones command of MiXCR [54]. The exported clonotypes were visualized in the form of a chord diagram using VDJtools software (version 1.1.10).

### Manufacture of clinical-grade ITNKs

For the clinical trial in this study, ITNKs were derived from autologous T cells that were engineered with CRISPR/Cas9 to knock out BCL11B. ITNKs were manufactured at a Good Manufacturing Practice (GMP) laboratory at Guangdong Zhaotai InVivo Co. Ltd. (GZI). An overview of the manufacturing process is shown in Fig. S10D. On day 0, autologous T cells were obtained by apheresis. Mononuclear leukocytes were isolated from the apheresis product by Ficoll density gradient centrifugation. T cells were enriched from mononuclear leukocytes with CliniMACS CD4 reagent (200–070-132, Miltenyi Biotec, Germany) and CliniMACS CD8 reagent (200–070-115, Miltenyi Biotec, Germany) and activated by MACS GMP T cell transaction (170–076-156, Miltenyi Biotec, Germany) for 72 h according the manufacture’s protocols. On day 4, T cells were electroporated (Amaxa Nucleofector 2b, Amaxa® Human T Cell Nucleofector® Kit, Lonza, Germany) with 3 μg sgRNA-*BCL11B* plasmids for 1 × 10^7^ cells. On day 5, sgRNA-*BCL11B*-transduced T cells were cultured in T551 H3 medium containing 5% CTS immune cell SR (A2596101, Thermo Fisher, USA), 500 U/ml rh-IL2, and gentamycin sulfate (20 μg/ml). On day 7, the cells were transferred to T175 flasks with daily perfusion, and cultures were allowed to continue expansion until the harvest day. On the harvest day, a small sample of cells was collected and screened for pathogenic microorganisms and contaminants (bacteria, fungi, virus, mycoplasma, and endotoxins). Another small sample of cells was collected for evaluation of the killing capacities of ITNKs against K562 cells in vitro. The rest of the cells were harvested, washed, formulated, and cryopreserved in infusible cryomedia. Small-scale cultures were included for mock electroporation controls and potency controls. Absolute cell counts were obtained during large-scale culture using a cell counter (Countstar®, BioMed, China).

### Clinical summaries of subjects

**Subject GD001,** 47 years old, was first diagnosed with tumor–node–metastasis (TNM) stage II nasopharyngeal carcinoma (NPC) in 2014. Initially, he was treated with local radiotherapy but refused chemotherapy. In 2016, he underwent surgical resection of the left parotid region due to metastasis and refused to undergo chemotherapy again. His right lower lung had a mass with lymph node metastasis in the right hilum and mediastinum, accompanied by right pleural effusion, in March 2019. His biopsy analysis confirmed metastasis from NPC prior to T cell harvest for ITNK cell therapy. The subject underwent lymphodepleting chemotherapy and received intravenous infusion of ITNKs three times: dose level 1 on 19 April 2019 (D0); dose level 1 on 23 April 2019 (D4); and dose level 1 on 29 April 2019 (D10). Because this subject was the first recipient of ITNKs, dose escalation was not performed. He did not experience infusion-related complications, CRS, or neurotoxicity. Since we found that approximately 50% of thawed ITNKs remained viable after the second infusion on 23 April 2019, we repeated the ITNK infusion on 29 April 2019. At days 28 and 60, the patient received staging evaluations revealing progressive disease. He expired on hospice care.

**Subject GD002**, 49 years old, was first diagnosed with TNM stage IIIB rectal carcinoma in 2015. She was first treated with 9 cycles of mFOLFOX6 adjuvant chemotherapy and local radiotherapy upon the last cycle of chemotherapy. She did well for the next 4 years. When she developed metastasis in the sacrum detected by PET-CT in March 2019, she joined the clinical trial. She underwent 1 cycle of chemotherapy with capecitabine and zoledronic acid. The subject underwent lymphodepleting chemotherapy and received intravenous infusion of ITNKs on 13 May 2019 (D0) at dose level 1 without severe infusion-related complications. This subject then received ITNKs intravenously twice at dose level 2 on 27 May 2019 (D14) and 27 June 2019 (D45). No obvious adverse effects were observed post infusion. This subject was then infused intravenously with ITNKs at dose level 3 twice on 20 July 2019 (D68) and 16 August 2019 (D95). She experienced slight fever and fatigue upon each cell infusion but did not suffer any CRS or neurotoxicity. Due to disease stability, a lack of adverse effects and the absence of other therapeutic options, the TMC agreed to long-term administration for this subject on a compassionate basis after dosage escalation. The patient received ITNKs once every 30 days at maintenance doses (Table 1). Once per month, the subject received staging evaluation. The subject had not received any other treatments except ITNK cell infusion.

**Table 1:** Summary of patients and clinical responses

Patient	Gender	Age	Histology	HLA-I scores	NCR ligands scores	% of ITNK cytotoxicity against K562	Route of administration	Date of infusion	Dose level	Doses (***×*** 10^6^/kg)	Toxicity	Responses	Response duration (Months)
GD001	M	47	NPC	2	5	61.5	I.V.	2019/04/19	1	2.26	None	Progress	NR
								2019/04/23	1	2.26			
								2019/04/29	1	2.54			
GD002	F	49	CRC	1	7	77.3	I.V.	2019/05/13	1	2.64	Slight fever, fatigue	Partial remissions	16
								2019/05/27	2	7.04			
								2019/06/27	2	5.28			
								2019/07/20	3	10.57			
								2019/08/16	3	8.61			
								2019/09/20	1	2.36			
								2019/10/9	2	3.46			
								2019/12/03	2	4.38			
								2020/01/07	3	12.12			
								2020/04/15	3	11.53			
								2020/06/03	3	9.56			
								2020/07/14	3	9.48			
								2020/08/19	3	9.48			
								2020/09/22	2	5.53			
GD003	M	42	Rhabdomyosarcoma	3	3	51.0	I.A. ^a^	2019/08/02	1	4.00	Slight fever, fatigue, muscle pain	Progress	NR
								2019/08/16	0	0.15			
								2019/08/29	1	1.33			
GD004	M	29	Melanoma	1	7	71.3	I.A.	2019/08/16	1	1.26	Slight fever, fatigue	Stable	1
GD005	F	61	Gastric cancer	1	7	75..9	I.V.	2019/08/28	0	0.31	None	Stable	1
GD006	F	28	AMC	1	6	74.0	I.A. ^b^	2019/06/09	1	1.09	None	Stable	3
							I.V.	2019/08/11	2	4.42			
GD007	M	63	NSCLC	NA	NA	91.1	I.V.	2019/10/24	1	3.32	Slight fever, fatigue	Stable	3
GD008	M	59	CRC	NA	NA	83.1	I.T. ^c^	2020/03/04	1	2.51	Slight fever, fatigue	Stable	1.5
							I.V.	2020/03/09	1	2.51			
GD009	M	68	Melanoma	NA	NA	15.1	I.V.	2019/08/27	1	2.37	Slight fever	Progress	NR
							I.V.	2019/10/01	2	3.36			

*I.V* intravenous, *I.A.*^a^ Hepatic arterial and ascending aortic injection, *I.A.*^b^ uterine artery injection, *I.T.*^c^ intra-tumour injected, Information of the seven patients in the ITNK clinical trial on gender, age, cancer type, HLA-I and NCR ligands expression in tumour biopsy, ITNK cytotoxicity, ITNK infusion dosage, toxicity, clinical outcomes and response duration. *NPC* nasopharyngeal carcinoma, *CRC* colorectal cancer, *AMC* appendices mucinous cystadenocarcinoma, *NSCLC* non-small cell lung carcinoma, *NA* not available, *NR* no response

**Subject GD003**, 42 years old, was first diagnosed with rhabdomyosarcoma in the left armpit in 2017. At first, he received 5 cycles of epirubicin, platinum and ifosfamide adjuvant chemotherapy. He developed liver, pancreas, lung and spine recurrence and was treated with radiofrequency ablation for the liver, high-intensity focused ultrasound (HIFU) for the pancreas, resection including the right lower lung and one vertebral resection and TomoTherapy radiation treatment for partial liver and lung metastases. After local therapies, he received apatinib, nivolumab and everolimus but experienced progression prior to T cell harvest. The subject underwent lymphodepleting chemotherapy and received hepatic arterial and ascending aortic injection of ITNKs at dose level 1 on 2 August 2019 (D0). After infusion, this patient experienced fatigue, slight fever, and muscle pain with elevated IL-6 in serum. Although no severe adverse effects were observed in this patient, as the first patient with an IL-6 spike in this clinical investigation, he was administered tocilizumab to prevent potential CRS. In particular, the IL-6 concentration in serum of GD003 (669.3 pg/ml), the patient with the highest IL-6 concentration in this study, was still lower than the highest IL-6 concentrations in anti-HER2 CAR-T recipients (1000 pg/ml) [55], in anti-IL13Rα2 CAR-T recipients (1062.5 pg/ml) [56], and in anti-CEA CAR-T recipients (above 1000 pg/ml) [57]. The subject recovered from CRS quickly and remained eligible for ongoing trial participation. He then received ITNKs at dose level 0 on 16 August 2019 (D14) without infusion-related complications. We repeated the infusion of ITNKs at dose level 1 on 29 August 2019 (D27). At days 30 and 60, his staging evaluations showed progressive disease according to the RECIST 1.1 criteria; however, his serum CA199 was decreased temporarily during the course of ITNK infusion. He was admitted to a local hospital for palliative care.

**Subject GD004**, 29 years old, was first diagnosed with TNM stage IV melanoma from an unknown site manifested as metastases in the neck and liver in 2017. He was first treated with 4 cycles of albumin–paclitaxel, bevacizumab and pembrolizumab with progression, followed by 4 lines of therapy including various combinations of temozolomide, dacarbazine, cisplatin, vindesine, semustine, ipilimumab, nivolumab and local microwave ablation or radiation, also with progression. He underwent steady-state T cell harvest prior to fludarabine and cyclophosphamide lymphodepleting chemotherapy before ITNK cell infusion. He received an intra-arterial infusion of ITNKs into the hepatic artery and ascending aorta on 16 August 2019 (D0) at dose level 1 without infusion-related complications. He experienced slight fever but no CRS or neurotoxicity. At day 30, staging evaluation revealed mixed response and stable disease according to the RECIST 1.1 criteria. At day 90, he met the criteria for progression and received surgery on his spine. He remains alive with progression but did not show any new or ongoing trial-related adverse events 14 months post ITNK cell infusion. He received treatment at a local hospital.

**Subject GD005**, 61 years old, was first diagnosed with TNM stage IV gastric carcinoma manifested by metastases in the omentum and ovaries in 2017. She was first treated with 6 cycles of oxaliplatin and tegafur/gimeracil/oteracil potassium adjuvant chemotherapy. She developed local recurrence and had not received active therapy for 3 months prior to T cell harvest and lymphodepleting chemotherapy and ITNK cell infusion. As ITNKs for this subject could not be manufactured at dose level 1, the ITNKs produced were given at dose level 0 intravenously on 28 August 2019 (D0), with the agreement of the TMC. She experienced no CRS or neurotoxicity. At day 28, her staging evaluation showed stable disease according to the RECIST 1.1 criteria. At day 60, she met the criteria for progression and started to receive other chemotherapy. The subject was treated at a local hospital.

**Subject GD006**, 28 years old, was first diagnosed with TNM stage III appendiceal mucinous cystadenocarcinoma identified during surgery in 2016. She was first treated with 1 cycle of oxaliplatin and 5-FU, followed by a second surgery and 3 rounds of intraperitoneal heat perfusion of oxaliplatin, irinotecan and raltitrexed. She progressed with local recurrence followed by surgery and 3 rounds of intraperitoneal heat perfusion of oxaliplatin, irinotecan and raltitrexed, followed by 4 cycles of Xeloda (capecitabine) with progression. She underwent steady-state T cell harvest. Before ITNK cell infusion, she received cyclophosphamide as lymphodepleting chemotherapy, which caused acute nausea and vomiting. Therefore, fludarabine administration was suspended for this patient. The subject underwent intra-arterial infusion of ITNKs into both uterine arteries at dose level 1 on 9 June 2019 (D0) without infusion-related complications. She then received intravenous infusion of ITNKs at dose level 2 on 11 August 2019 (D63) without infusion-related complications. She did not experience any CRS or neurotoxicity. At days 28, 60 and 90, staging evaluations revealed stable disease according to the RECIST 1.1 criteria. However, at day 120, she had progressive disease and was begun on PD-1 antibody and bevacizumab with progression. She is now on PD-1 and CTLA-4 antibodies and bevacizumab and has shown an initial response; she also underwent a third debulking surgery in the abdomen. She remains alive without new or ongoing study-related adverse events post ITNK cell infusion.

**Subject GD007**, 63 years old, was first diagnosed with TNM stage IV nonsmall cell lung adenocarcinoma in his right upper lung in 2017. He was first treated with erlotinib but experienced progression and was then treated with osimertinib. He developed EGFR-TKI resistance and was treated with 4 cycles of chemotherapy, including pemetrexed, carboplatin and apatinib, with continued progression. He had not received active therapy for 3 months prior to T cell harvest for ITNK cellular immunotherapy. The subject underwent lymphodepleting chemotherapy and received intravenous infusion of ITNKs at dose level 1 on 24 October 2019 (D0) without any infusion-related complications. He experienced slight fever and fatigue without CRS or neurotoxicity. At day 28, staging evaluation showed mixed response and stable disease according to the RECIST 1.1 criteria; in January 2020, disease progression was noted and was followed by other unknown therapies. His disease is stable, and he had no new or ongoing study-related adverse events 90 days post ITNK infusion.

**Subject GD008**, 59 years old, was first diagnosed with TNM stage IV rectal adenocarcinoma manifested by multiple metastases in the liver and abdominal lymph nodes in 2019. He was first treated with 9 cycles of mFOLFOX6 plus bevacizumab and local radiotherapy upon the fifth cycle of chemotherapy. He progressed and received transarterial chemoembolization twice for liver metastases. After lymphodepleting chemotherapy, the subject received an intratumoral injection of ITNKs into three liver lesions at dose level 1 on 4 March 2020 (D0). Because this subject was the first intratumoral recipient of ITNKs, dose escalation was not performed. He received an intravenous infusion of ITNKs at dose level 1 on 9 March 2020 (D5). He experienced slight fever and fatigue upon the first 2 days of cell infusion but no CRS or neurotoxicity. At day 45, staging evaluation revealed stable disease according to the RECIST 1.1 criteria. However, the second harvest of PBMCs failed, and he had to leave the trial to seek other therapies.

**Subject GD009**, 68 years old, was diagnosed with TNM stage IIIB melanoma from the upper palate mucosa manifested by invasion of the palate bone in 2017. He was first treated with 4 cycles of dacarbazine, cisplatin and bevacizumab with progression followed by 2 lines of therapy, including oxaliplatin, epirubicin and ipilimumab, with progression. The patient underwent T cell harvest prior to fludarabine and cyclophosphamide lymphodepleting chemotherapy before ITNK cell infusion. He received a venous infusion of ITNKs at dose level 1 on 27 August 2019 (D0) without infusion-related complications. He then received intravenous infusion of ITNKs at dose level 2 on 1 October 2019 (D35) without infusion-related complications. He experienced slight fever and no CRS or neurotoxicity. At day 45, staging evaluation revealed progressive disease according to the RECIST 1.1 criteria. He was begun on other chemotherapy with albumin–paclitaxel and carboplatin and radiotherapy outside of this trial.

### Statistic

Statistical significance was determined using Student’s t test (two groups) or ANOVA with Tukey’s multiple comparison test (three or more groups). Survival was plotted using a Kaplan-Meier survival curve, and statistical significance was determined by the log-rank (Mantel-Cox) test. All statistical analyses were performed using Prism software, version 7.0 (GraphPad, Inc., San Diego, CA, USA). Statistical significance was indicated at *P ≤ 0.05, ** *P* ≤ 0.01, and ***P ≤ 0.001.

## Results

### Reprogramming human cord and peripheral blood T cells into ITNKs

**Fig. 1:**
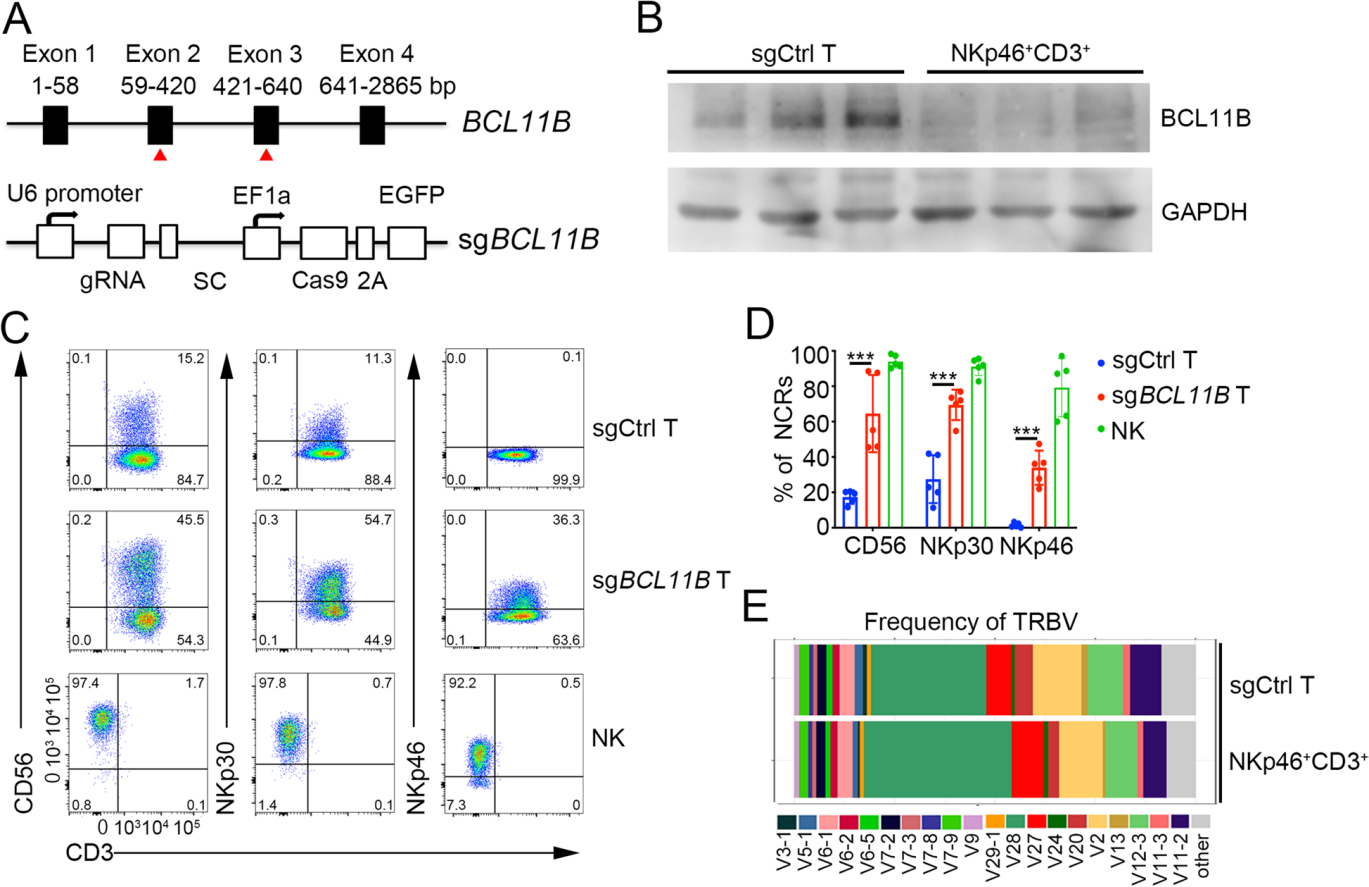
Reprogramming of primary human T cells into ITNKs by inactivating BCL11B. **A** sgRNA targeting exon 2 and exon 3 of the *BCL11B* locus. sgRNA, Cas9 and EGFP elements were integrated into a single vector. **B** Western blot analysis of BCL11B (120 kDa) levels in three representative samples of CB-derived T cells that were transduced with sgCtrl or NKp46^+^CD3^+^ cells (purity: 92.41 ± 2.60%) that were sorted from sg*BCL11B*-engineered T cells. **C** Representative flow cytometric detection of CD3, CD56, NKp30 and NKp46 in T cells: T cells transduced with sgCtrl, T cells transduced with sg*BCL11B* and normal NK cells (CD3^−^CD56^+^). Data are representative of five independent experiments. **D** Graph summarizing the percentages of CD56^+^, NKp30^+^, and NKp46^+^ cells in CD3^+^ T cells that received sg*BCL11B* or sgCtrl at 14 days post electroporation. The mean values of five independent healthy donors are shown. *P* < 0.001 for CD56^+^, NKp30^+^ and NKp46^+^ T cells in sg*BCL11B*-electroporated T cells compared to sgCtrl-electroporated T cells. ***P ≤ 0.001, two-way ANOVA with Sidak’s multiple comparisons test. **E** TCR diversity in sgCtrl T and NKp46^+^CD3^+^ cells purified from sg*BCL11B*-edited T cells from the same donor based on variable chain sequencing data for the TCRβ locus. The 20 variable chain sequences at the TCRβ locus were analyzed

To inactivate *BCL11B*, we transfected human cord blood (CB) T cells with a plasmid expressing Cas9-EGFP and sgRNAs targeting either exon 2 or 3, alone or in combination (Figs. 1A and S1A). Cells transfected with both sgRNA and Cas9 cultured under T cell conditions showed limited cell death (Fig. S1B) and still expressed GFP 5 days after electroporation (Fig. S1C), and up to 47.2% of the cells had become NKp46 positive at Day 14 (Fig. S1D). DNA sequencing confirmed gene editing at the *BCL11B* locus, including frameshifts in exons 2 and 3 (Fig. S1E), and western blot analysis revealed a loss of the BCL11B protein in NKp46^+^CD3^+^ cells (Fig. 1B). In the experiments presented below, we used two sgRNAs targeting exons 2 and 3 of *BCL11B* (sg*BCL11B*) to increase the efficiency of *BCL11B* gene editing. In addition to increasing expression of NKp46, sg*BCL11B*-transduced T cells also upregulated the NK cell markers CD56 and NKp30 while retaining expression of the T cell marker CD3 (Fig. 1C, D). Conversely, T cells expressing an unrelated sgRNA (sgCtrl) lacked NKp46 expression (Fig. 1C, D). NKp46^+^CD3^+^ cells were viable, as verified by imaging flow cytometric analysis (Fig. S1F). The T cell origin of NKp46^+^CD3^+^ cells was confirmed by analyzing their TCRβ repertoire and comparing it to that of polyclonal T cells from the same donor (Fig. 1E). Compared to sgCtrl-transduced T cells, sg*BCL11B*-transduced T cells did not show an effect on growth through 21 days post electroporation (Fig. S1G). In addition to their derivation from CB T cells, ITNKs could be efficiently generated by ablating BCL11B in peripheral blood T cells (Fig. S2A-B). Since cell populations derived from either CB or peripheral blood mononuclear cells (PBMCs) contain various types of lymphocytes, including both CD4^+^ T cells and CD8^+^ T cells, we investigated the cellular heterogeneity of ITNKs. A subset of ITNKs from CB or PBMCs were also either CD8^+^ or CD4^+^, with CD8-expressing cells being more frequent (Fig. S2C, D). CD4^+^ ITNKs strongly expressed CD56 and NKp30 (Fig. S2C, D). BCL11B deletion did not affect the survival or expansion of CD8^+^ and CD4^+^ T cells during 3 weeks of culture (Fig. S2E). Furthermore, upon BCL11B loss, memory (CD45RA^−^/CD45RO^+^) and effector (CD45RA^+^/CD45RO^−^) CD4^+^ and CD8^+^ T cells were reprogrammed into NKp30^+^CD3^+^CD4^+^ and NKp46^+^CD3^+^CD8^+^ ITNKs, respectively (Fig. S3A). Of interest, the percentages of ITNKs in CD8^+^ and CD4^+^ effector T cell cultures were higher than those in CD8^+^ and CD4^+^ memory T cell cultures, respectively (Fig. S3A), suggesting that effector T cells reprogram into ITNKs more efficiently than do memory T cells after BCL11B ablation. In addition, we detected γδTCR^+^ ΙTNKs with a γδTCR^+^NKp30^+^ phenotype (Fig. S3B) and mucosal-associated invariant T (MAIT)-derived ITNKs, which expressed both TCRvα7.2, a MAIT cell marker [58], and NKp30 (Fig. S3C), following BCL11B inactivation. We therefore surmise that acute loss of BCL11B reprograms major human T cell subtypes, including CD8^+^ T, CD4^+^ T, γδ T, and MAIT cells, into ITNKs.

### Immunophenotypic characteristics of ITNKs

**Fig. 2:**
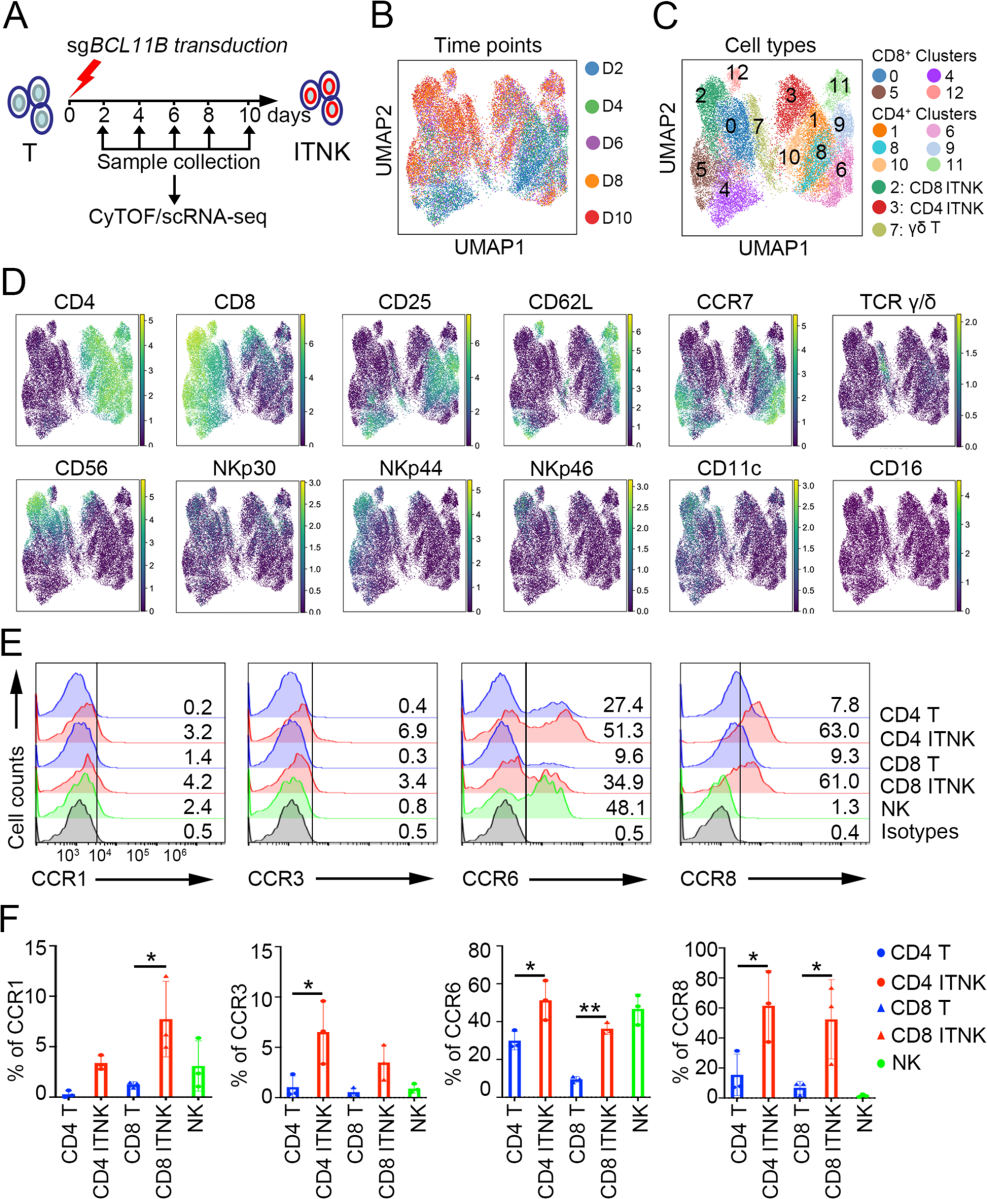
Immunophenotypic characteristics of ITNK cells. **A** Representative CyTOF analysis of the immunophenotypic profiles of CB-derived T cells on Day 2 to Day 10 post electroporation with sg*BCL11B*. **B, C** UMAP plots with colored circles highlighting the cells at different time points (Day 2, Day 4, Day 6, Day 8, and Day 10) (**B**) and different clusters of T cells based on their immunophenotypic profiles (**C**). **D** Representative T cell marker (CD4, CD8A, CD62L, CCR7, and γδTCR) and NK cell-associated marker (CD56, NKp30, NKp44, NKp46, CD11c and CD16) expression in various subtypes of T cells and ITNKs. **E** Representative flow cytometric detection of CCR1, CCR3, CCR6, CCR8, and CXCR4 in T cells (CD3^+^CD4^+^/CD8^+^), ITNKs (CD3^+^CD4^+^NKp30+/CD3^+^CD8^+^NKp46^+^) and normal NK cells (CD3^−^CD56^+^). Data are representative of three independent experiments. **F** Graph summarizing the percentages of CCR1, CCR3, CCR6, CCR8, and CXCR4 cells in T cells, ITNKs and NK cells after 14 days of culture. The results represent the mean ± SD. *P ≤ 0.05, **P ≤ 0.01, and ***P ≤ 0.001; one-way ANOVA with Tukey’s multiple comparisons test

We next investigated the time-dependent immunophenotypic dynamics of the T cell-to-ITNK transition at the single-cell level using cytometry by time of flight (CyTOF) (Fig. 2A). Projection of CyTOF-measured expression into UMAP space allowed time-resolved visualization (D2-D10) of ITNK reprogramming (Fig. 2B) and identified 13 clusters of T cells (Fig. 2C), revealing that T cells in Clusters 0, 2, 4–5, and 12 expressed CD8, while those in Clusters 1, 3, 6, 8, and 9–11 were CD4 T cells (Fig. 2C-D). NK cell-associated markers (CD56, NKp30, NKp44, and NKp46) were mainly expressed in Clusters 2 and 3, suggesting that these clusters corresponded to CD8 and CD4 ITNKs, respectively (Fig. 2C-D). γδ ΙTNKs were detected in Cluster 7 (Fig. 2C-D). Interestingly, CD11c, a marker of dendritic cells [59] that is suppressed by Bcl11b in mouse T cells [20], was specifically upregulated in CD8 ITNKs (Cluster 8) compared to cells in Clusters 4, 5 (Figs. 2C-D, S4A). Some inhibitory receptors (PD1 and TIGIT) were expressed at low levels, while CTLA4 and TIM3 were highly expressed in CD8 ITNKs (Fig. S4A). However, unlike NK cells, neither ITNKs nor T cells expressed inhibitory NK cell receptors, including CD158b, CD158e1, CD94, and NKG2A [60] (Fig. S4B, C). Infiltration of T and NK cells into tumors is crucial for tumor clearance [61–63]. We thus characterized the expression profiles of chemokine receptors and found that CCR1, CCR3, CCR6, and CCR8 were all upregulated in ITNKs compared to T cells (Fig. 2E, F). Taken together, our data show that ITNKs maintain the expression of T cell surface markers and acquire NK cell-associated activating receptors.

### ITNKs acquire transcriptional profiles of NK cells

**Fig. 3:**
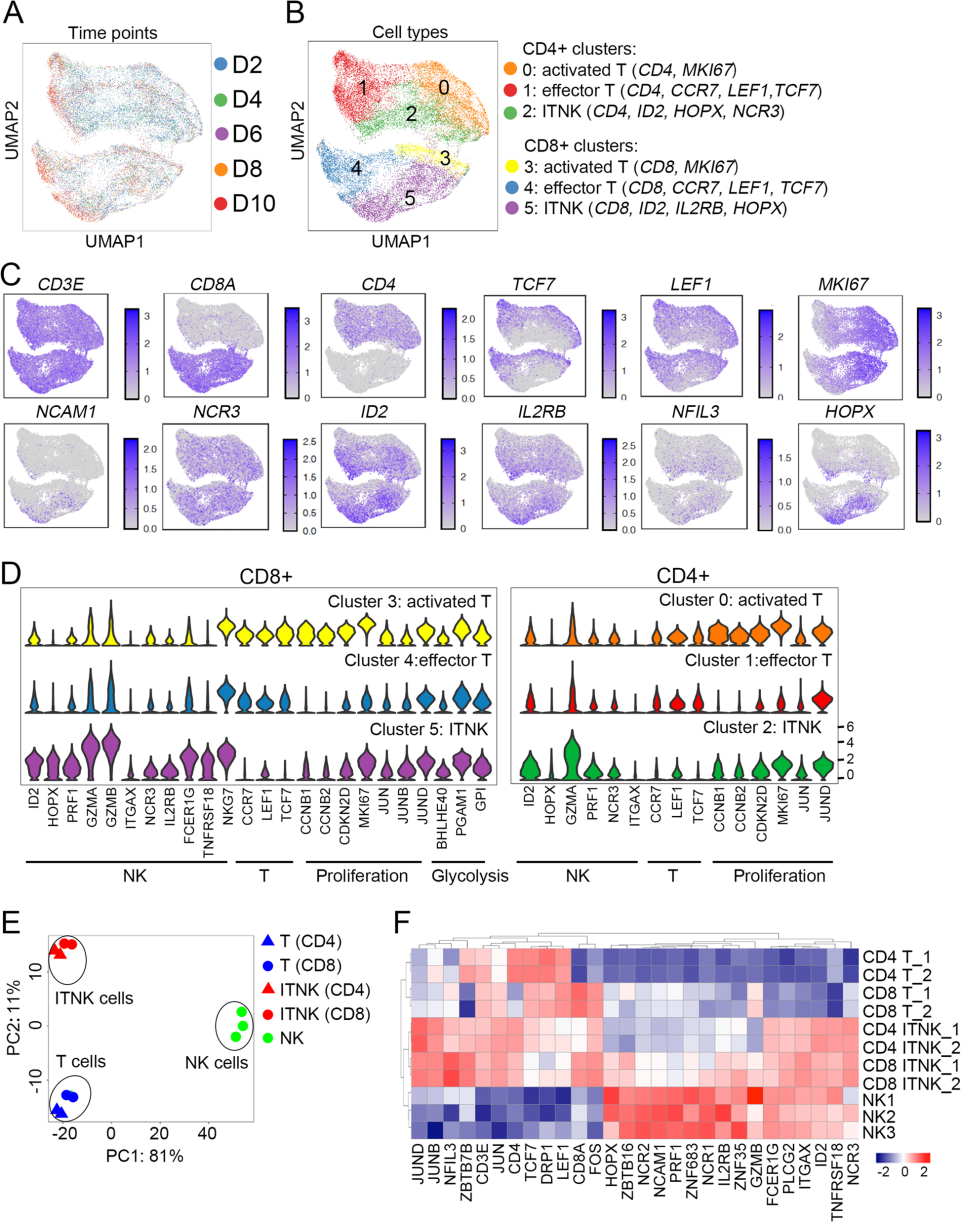
ITNKs acquire transcriptional profiles of NK cells. **A** UMAP visualization of sg*BCL11B*-transduced T cells after batch correction, colored by samples collected for scRNA-seq analysis on Day 2 (D2), Day 4 (D4), Day 6 (D6), Day 8 (D8), and Day 10 (D10) post electroporation. **B** UMAP visualization of sg*BCL11B*-transduced T cells mainly classified into 6 different clusters based on their gene expression profiles. **C** Plots showing the expression levels of selected genes associated with T (C*D3E, CD8A* and *CD4*) or NK cell lineages (*NCAM1, NCR3, ID2, IL2RB,* and *NFIL3*) in sg*BCL11B*-transduced T cells based on scRNA-seq analysis. **D** Violin plots showing the expression levels of NK cell- and T cell-associated genes, AP-1 family genes, glycolysis-associated genes and genes regulating proliferation in activated T cells (Cluster 0 (CD4 T) and Cluster 3 (CD8 T)), effector T cells (Cluster 1 (CD4 T) and Cluster 4 (CD8 T)), and ITNKs (Cluster 2 (CD4 ITNK) and Cluster 5 (CD8 ITNK)). **E** T cells and ITNKs were purified (purity > 90%) from sgCtrl- and sg*BCL11B*-electroporated T cells from Donors 1 and 2. Purified NK cells (CD3^−^CD56^+^) (purity > 90%) were enriched from NK cell cultures from Donors 1–3. Cells from Donors 1–3 were collected from CB samples. Principal component analysis (PCA) was used to evaluate the similarities in global gene expression profiles among purified ITNKs, NK cells, and T cells. **F** Differential expression patterns of selected NK cell-, glycolysis-, and T cell-associated genes and AP-1 family genes differentially expressed in ITNKs, NK cells, and T cells based on RNA-seq analysis

We further performed time-resolved scRNA-seq analysis of the same samples evaluated by CyTOF and identified three CD8^+^ clusters and CD4^+^ clusters (Fig. 3A, B). Analysis of the expression profiles of T cell-associated surface markers (CD3, CD8A, and CD4) and transcription factors (*TCF7* and *LEF1)*_,_ and the NK cell-associated genes (*NCAM1*, *NCR3*, *ID2*, *IL2RB*, and *NFIL3)*, [64, 65] revealed that CD8^+^ ITNKs were identified mainly in Cluster 5, whereas CD4^+^ ITNKs were enriched in Cluster 2 (Fig. 3B, C). At the individual gene level, violin plot analysis further confirmed the upregulation of NK cell-associated genes and the concomitant downregulation of T cell-related genes in Clusters 5 and 2 compared to other clusters of CD8 T cells and CD4 T cells, respectively (Fig. 3D), which were consistent with the CyTOF analysis (Fig. S4A). Other highly upregulated genes in ITNKs included cell proliferation-related genes (*CCNB1*, *CCNB2*, and *CDKN2D*) [66] and AP-1 family members (*JUN*, *JUNB,* and *JUND*), which regulate T cell activation and proliferation [67] (Fig. 3D).

To further investigate the ITNK gene expression signature, we conducted bulk RNA-seq analysis of T cells (CD3^+^), ITNKs (CD3^+^CD4^+^NKp30^+^ and CD3^+^CD8^+^NKp30^+^) and NK cells (CD3^−^CD56^+^) derived from PBMCs. Principal component analysis (PCA) and unsupervised hierarchical clustering analysis revealed that ITNKs exhibited global transcriptomic features of both T cells and NK cells (Fig. 3E, F). Although ITNKs still expressed genes associated with TCR signaling, in general, they downregulated *TCF7* and *LEF1* (Fig. 3F and Tables S1, S2, S3), which was consistent with our scRNA-seq analysis (Fig. 3C, D). In addition to NCRs, genes encoding key NK cell-associated transcription factors, including *ID2*, *ZBTB16*, *NFIL3*, and *ZNF35* (the human homolog of *Zfp105*), [68], exhibited increased expression in ITNKs compared to T cells (Fig. 3F). KEGG analysis of the upregulated differentially expressed genes (DEGs) showed that NK cell-associated pathways (e.g., natural killer cell-mediated cytotoxicity and the Fc epsilon RI signaling pathway) were enriched in ITNKs compared to T cells (Tables S1, S2, S3), which is in line with previous findings showing that NK cell-associated genes are upregulated in Bcl11b-deficient murine T cells [16]. Thus, these analyses show that ITNKs acquire expression of NK cell-associated genes at the transcriptional level.

### ITNKs recognize and selectively lyse tumor cells

**Fig. 4:**
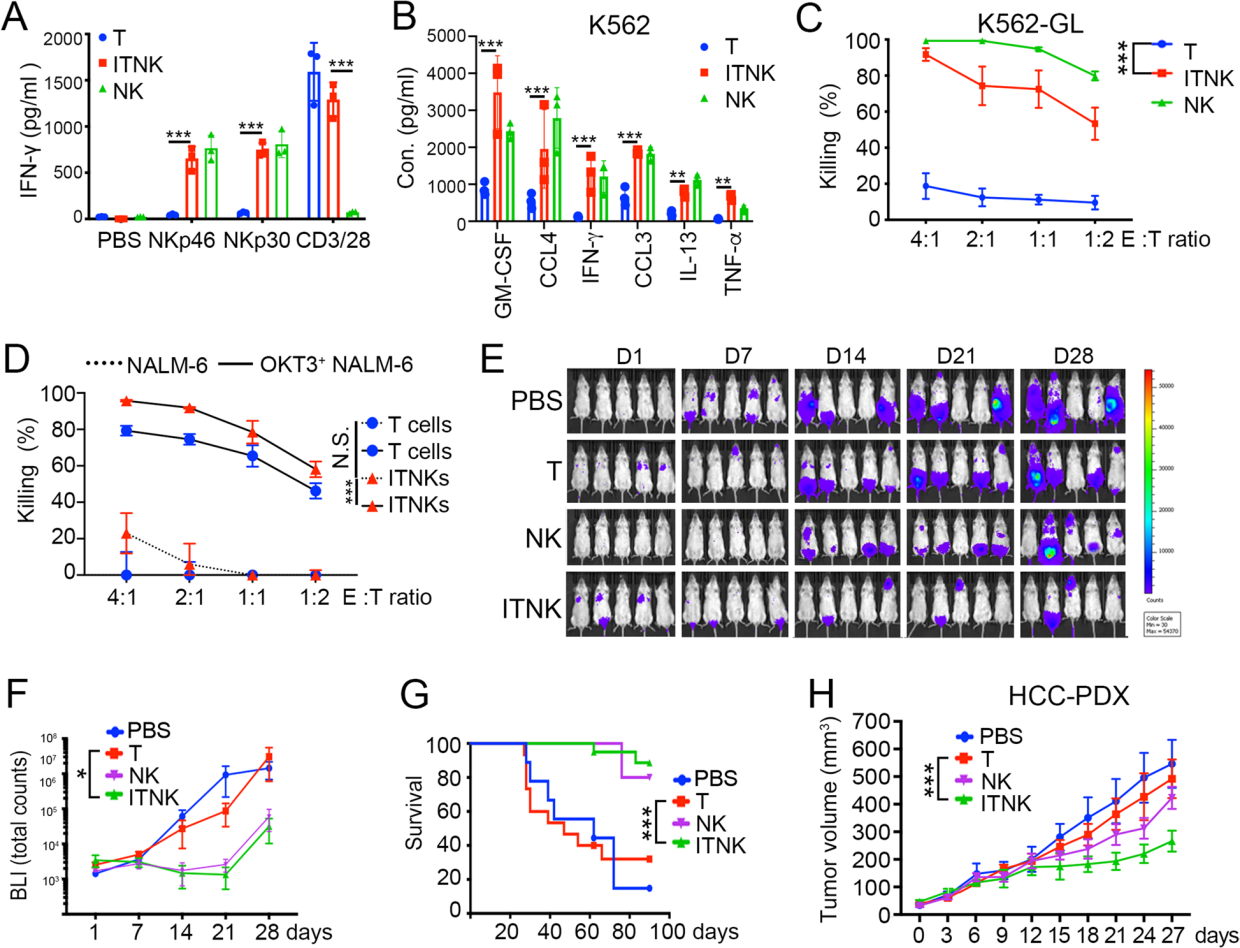
Evaluating the antitumor effects of ITNKs in vitro and in vivo. **A** The bar chart shows IFN-γ secretion by T cells, ITNKs and NK cells after activation for 24 h with the indicated antibodies (anti-NKp30, anti-NKp46 and anti-CD3/CD28 at 5 μg/ml). Samples were collected from three individual donors. Data are shown as the mean ± SD; *P ≤ 0.05 and ***P ≤ 0.001; two-way ANOVA with Tukey’s multiple comparisons test. **B** Cytokine secretion profiles of ITNKs, T cells, and NK cells in coculture with K562 cells. ITNKs, T cells, and NK cells were incubated with K562 cells at an E:T ratio of 1:1 for 24 h. The supernatants were then harvested, and the concentrations of the indicated cytokines were measured by a multiplex immunoassay. The values shown represent the mean ± SD of 3 different donors. **P ≤ 0.01 and ***P ≤ 0.001; two-way ANOVA with Tukey’s multiple comparisons test. **C** Killing assays showing the percent cytotoxicity of ITNKs, NK cells and T cells against K562 cells. The data are shown as the mean ± SD; *P* < 0.0001 (ITNKs vs. T cells for K562 cells) and ***P ≤ 0.001; two-way ANOVA with Tukey’s multiple comparisons test. **D** The percent cytotoxicity of ITNKs and T cells against NALM-6 and OTK3-overexpressing NALM-6 cells in coculture. The results were obtained from three independent experiments. *P* < 0.0001 (ITNKs lysing OKT3^+^ NALM-6 cells vs. ITNKs lysing NALM-6 cells) and ***P ≤ 0.001; two-way ANOVA with Tukey’s multiple comparisons test. **E** Bioluminescence images showing the fate of K562 cells transplanted into NSI mice at the indicated time points. **F** Quantification of the total flux analyzed by in vivo bioluminescence imaging of luciferase activity (*n* = 5, per group). The results represent the mean ± SD; *P* = 0.0347 (ITNKs vs. T cells) and *P ≤ 0.05; two-way ANOVA with Tukey’s multiple comparisons test. **G** Survival analysis of mice treated with PBS (*n* = 10), T cells (*n* = 15), ITNKs (*n* = 15), or NK cells (*n* = 5); *P* = 0.0001 (ITNKs vs. T cells) by the log-rank Mantel-Cox test. **H** Tumor progression of patient-derived HCC xenografts (donor: 47-year-old female with grade IV hepatocellular carcinoma) treated with PBS, T cells, ITNKs, or NK cells (*n* = 5). Data are shown as the mean ± SD; *P* = 0.0001 (ITNKs vs. T cells) by two-way ANOVA with Tukey’s multiple comparisons test, ***P ≤ 0.001

We next characterized ITNK cell functions. ITNKs secreted IFN-γ upon stimulation with anti-CD3/CD28, anti-NKp30, or anti-NKp46 antibodies (Fig. 4A). In contrast, T cells and NK cells were only responsive to anti-CD3/CD28 or anti-NKp30/NKp46 treatment, respectively (Fig. 4A). Upon coculture with K562 cells, an erythroleukemia cell line that lacks HLA-I expression [69], ITNKs were induced to secrete proinflammatory cytokines, including GM-CSF, IFN-γ and TNF-α (Fig. 4B), and lyse K562 cells (Fig. 4C). Of note, CD8^+^ ITNKs lysed K562 cells more potently than did CD4^+^ ITNKs (Fig. S5A), but the latter secreted more TNF-α (Fig. S5B). ITNKs also efficiently killed SK-OV-3 and HeLa cells, two human cell lines that strongly express NCR ligands [70] (Fig. S5C, D). Anti-HER2 antibodies further enhanced NK cell- but not ITNK cell-induced lysis of SK-OV-3 cells, suggesting that ITNKs do not acquire the ability to perform antibody-dependent cell-mediated cytotoxicity (ADCC) against tumor cells (Fig. S5C), in line with their lack of CD16 expression (Fig. 2D). The killing of HeLa cells by ITNKs was partially repressed by blocking NKp30 or NKp46 (Fig. S5D), suggesting that ITNKs recognize tumor targets through these two receptors. In contrast to their effects on K562, SK-OV-3, and HeLa cells, ITNKs exhibited only limited cytotoxicity to pre-B cell leukemia NALM-6 cells, which matched the low expression of NCR ligands and high expression of HLA-I molecules in NALM-6 cells [71] (Fig. 4D). However, ITNKs efficiently lysed OKT3^+^ NAML-6 cells that ectopically expressed OKT3, an anti-CD3 single chain fragment variation (scFv) that stimulates TCR [72] (Fig. 4D), suggesting that ITNKs were still able to recognize targets through their TCR. It has been shown that patient-derived tumor organoids (PDOs) can recapitulate the pathophysiology of primary tumors [73]. We found that both ITNKs and NK cells efficiently killed hepatocellular carcinoma (HCC) PDOs (Fig. S5E). These findings show that ITNKs exhibit tumor cell-killing activity in vitro and indicate that ITNKs recognize tumor targets through both TCR and NCR.

We next evaluated the antitumor effects of ITNKs in xenograft models. ITNKs and T cells shared similar expression profiles for CD45RA and CD45RO (Fig. S5F and Table S4). K562 cells expressing luciferase for bioluminescence imaging (BLI) were inoculated into immunocompromised mice (NSI: NOD/SCID/IL2RG^−/−^) [74], followed by infusion of ITNKs, NK cells, or T cells (Fig. S5G). ITNK or NK cell infusion caused a significant decrease in the tumor burden measured by BLI 28 days after K562 cell inoculation (Fig. 4E, F) and prolonged survival (Fig. 4G). Notably, ITNK transplantation did not cause any detectable symptoms of graft-versus-host disease (GVHD) up to 3 months after infusion, as the GVHD scores [75] of the ITNK and NK groups remained at zero (Fig. S5H). Similar results showing ITNK-mediated suppression of tumor cell xenografts were obtained for solid tumor cells (human primary HCC tumor cells in Fig. 4H and HeLa cells in Fig. S5I, J). Human ITNKs were therefore found to be effective in inhibiting both blood-derived and solid tumors in the xenograft models tested.

### CAR improves the cytotoxicity of ITNKs against tumors

**Fig. 5:**
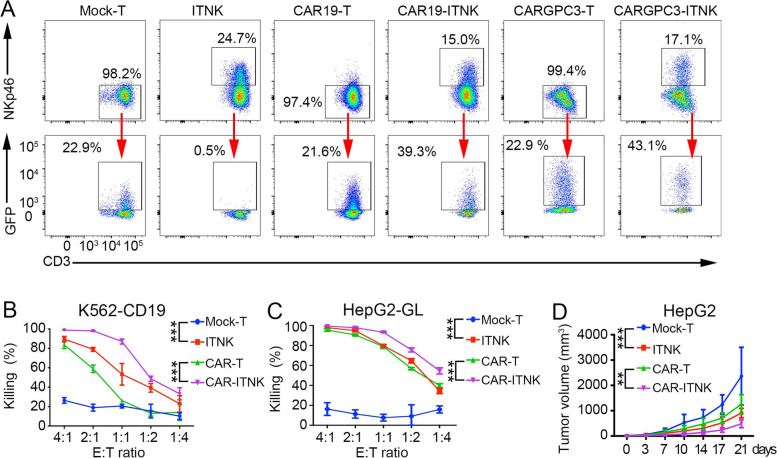
Enhanced anti-tumour activity in ITNKs engineered with CAR. **A** Representative flow cytometer analysis of CAR-ITNKs (CAR19-ITNKs and CARGPC3-ITNKs) which were defined as GFP + CD3 + NKp46+ on day 14 post electroporation; (**B**) ITNKs and T cells expressing CAR19 were co-cultured with K562 cells that expressed CD19 with the indicated ratios of effectors to targets. The percentage means of specific tumour cell lysis ± SD are shown. *P* < 0.0001 (CAR19-ITNK vs. CAR19-T) and *P* < 0.0001 (CAR19-ITNK vs. ITNK); ***P ≤ 0.001; two-way ANOVA with Tukey’s multiple comparisons test; (**C**) ITNK and T cells expressing CARGPC3 were co-cultured with HepG2-GL with the indicated effector to target ratios. The means of percentages of specific tumour cell lysis ± SD are shown. *P* < 0.0001 (CARGPC3-ITNK vs. CARGPC3-T), ***P ≤ 0.001; two-way ANOVA with Tukey’s multiple comparisons test; (**D**) Tumour progression of HCC xenografts treated with T, ITNK, CARGPC3-T and CARGPC3-ITNK cells (*n* = 5, per groups) on day 0 and day 3 when the volumes of tumours were about 50mm^3^. Data are shown as mean ± SD; *P* < 0.0001 (ITNK vs. T) and *P* = 0.0015 (CARGPC3-ITNK vs CARGPC3-T); two-way ANOVA with Tukey’s multiple comparisons test, **P ≤ 0.01 ***P ≤ 0.001

To improve the specificity of ITNK recognition of tumors, we transduced ITNKs with a CAR consisting of an anti-CD19 or anti-GPC3 scFv, a CD28 transmembrane domain and endodomain, a TLR2 endodomain [76] and a CD3ζ signaling domain using the piggyBac transposon system [77] or a lentivirus for stable expression (Fig. S6A, B). Up to 43.1% of ITNKs were transduced with the PB-CAR constructs (Fig. 5A). Since ITNKs were derived from T cells and retained high expression of low-density lipid receptors (LDLRs) (Fig. S6C), an essential receptor of the vesicular stomatitis virus G protein (VSV-G) of lentiviruses [78], the lentiviral transfection efficiencies in ITNKs were as high as 40% following only one round of transfection, while only 2.8% of NK cells were transfected (Fig. S6D).

ITNKs expressing CAR-CD19 (CAR19-ITNKs) were assessed for their antitumor efficacy against K562 cells engineered to express both human CD19 (K562-CD19) and luciferase. CAR19-ITNKs lysed K562-CD19 cells more efficiently than did CAR19-T cells or ITNKs (Fig. 5B). In contrast, CAR19-ITNKs and CAR19-T cells showed similar killing efficacies for NALM-6 cells (Fig. S6E), while ITNKs did not kill this cell line (Fig. 4D and Fig. S6E). Furthermore, introducing a CAR-GPC3 construct into ITNKs (CARGPC3-ITNKs) enhanced cytotoxicity against HepG2 human HCC cells [79] (Fig. 5C).

Engineered ITNKs were also evaluated in vivo by injecting K562-CD19 or HepG2 cells into immunocompromised NSI mice, followed by administration of CAR-ITNKs, ITNKs, CAR-T cells, or T cells (Fig. S6F). CAR19-ITNKs (Fig. S6G, H) and CARGPC3-ITNKs (Fig. 5D) demonstrated substantially more potent antitumor activities than CAR-T cells in the xenograft models. Therefore, engineering ITNKs with CAR molecules targeting specific tumor antigens further enhances the cytotoxicity of ITNKs, as CAR signaling and NCR signaling have been shown to synergistically achieve target cell recognition and cytotoxicity [80].

### ITNKs as a potential cell source for treating refractory and advanced solid tumors

The potent antitumor capacity of human ITNKs encouraged us to explore their potential in preclinical and clinical applications. We first examined the distribution of human ITNKs in samples of peripheral blood, spleen, bone marrow, liver, and lung of immunocompromised NSI mice taken on days 1, 7, 14, 21 and 180 post ITNK infusion (Fig. S7A). ITNKs persisted for 2 to 3 weeks in the recipients but were undetectable 180 days post infusion (Fig. S7B). The presence of ITNKs did not appear to cause GVHD, as mentioned above (Fig. S5H). We next carefully evaluated possible off-target genetic changes induced by the *BCL11B*-CRISPR strategy by whole-genome sequencing of purified CD3^+^NKp46^+^ ITNKs derived from two individual donors (Fig. S7C). A computational tool predicted 669 potential off-target sites with 3 mismatches and 1 bulge size [81, 82]. After excluding candidate sites with no insertions or deletions (indels) near the predicted off-target sites in ITNKs, we analyzed 16 candidate sites, including the two on-target loci (Table S5). Whole-genome sequencing results showed no indels in the other 14 candidates off-target sites (Table S5).

Encouraged by these results, we designed a clinical investigation to assess the safety and efficacy of infusing autologous ITNKs in patients diagnosed with advanced and metastatic cancer who did not respond to multiple prior therapies (Tables 1 and S6). Autologous ITNKs were manufactured from the PBMCs of nine patients. The manufacturing process and clinical protocol (NCT03882840) for the ITNKs and the CONSORT diagram are depicted in Figs. S7D, S8, and S9. The median age of the patients was 49.6 years, and six of the patients were men (Table 1). Tumor biopsies from patients GD001-GD006 were available and evaluated for the expression of HLA-I molecules that suppress NK cell activation [83] or for NCR ligands, which stimulate NK cytotoxicity against tumor cells [84] (Fig. S10A). Autologous ITNKs were manufactured from the PBMC fraction of the blood from the nine patients. All products were subjected to detailed release criteria testing (Table S7). We also sequenced the ITNKs from the 9 patients and applied Cas-OFFinder to predict potential off-target sites [85] (Fig. S10B). The median frequency of mutations, all of which were intron variants, at the 20 off-target sites tested was 0.1% (range, 0–1%). In contrast, the median frequency of mutations at the *BCL11B* locus, including intron variants, frameshifts, and in-frame deletions, was 9.8% (range, 7.0–12.0%) (Fig. S10C). ITNKs were cultured for up to 21 days with a 3- to 76-fold expansion while they showed no long-term growth prior to infusion (Table S8). ITNKs used for this trial, defined as CD3^+^NKp30^+^ cells, comprised 17.2–58.1% of all cells (Fig. S11A and Table S8) and were capable of efficiently lysing K562 cells in vitro (Table 1 and Fig. S11B).

**Fig. 6:**
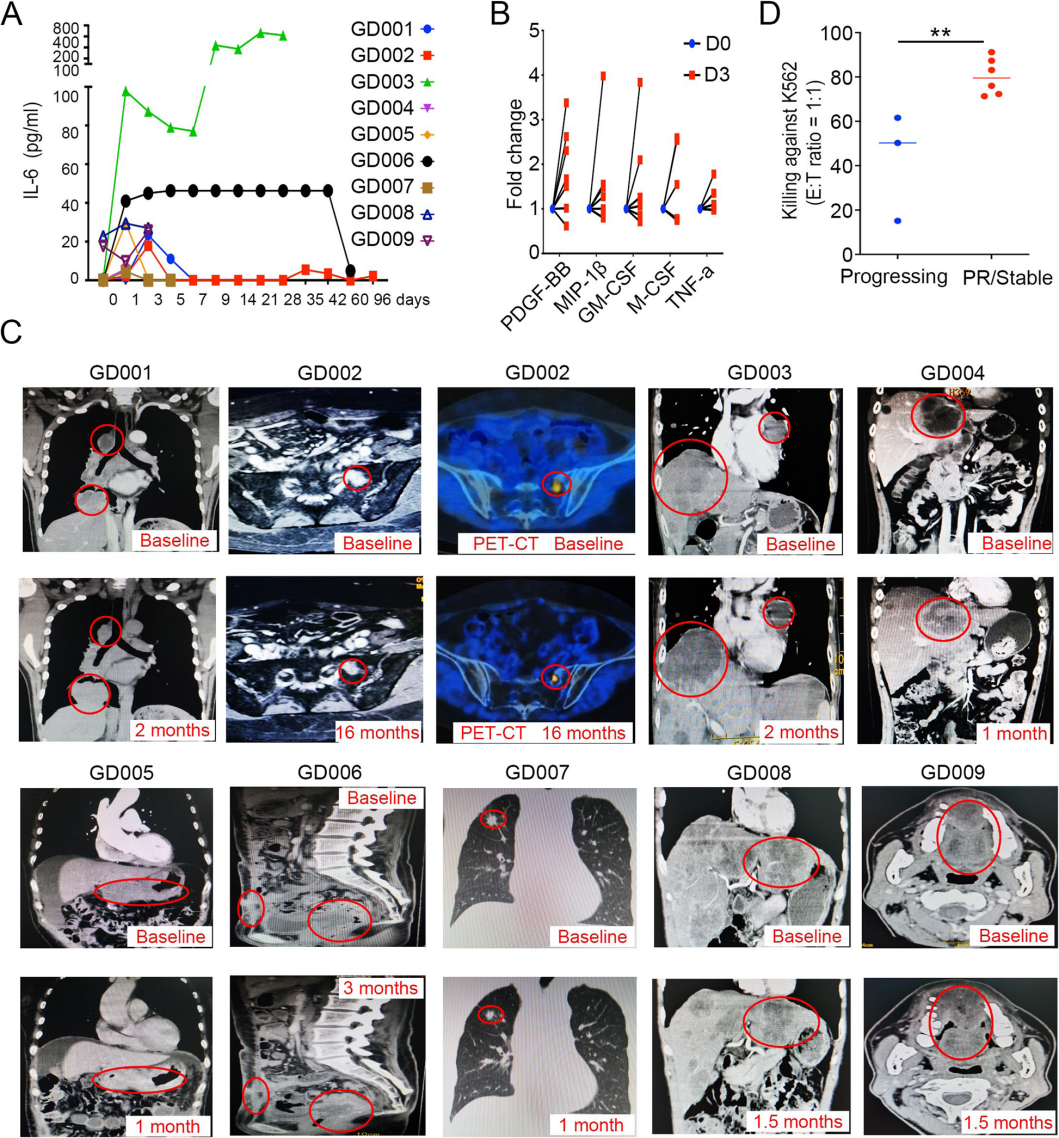
Safety and potency of ITNK infusions in cancer patients. **A** Serum IL-6 levels in patients (GD001-GD009) from day 1 to 3 months post ITNK infusion. **B** Cytokine levels in patients on day 0 and day 3 post ITNK infusion. PDGF-BB, MIP-1β, GM-CSF, M-CSF and TNF-α were significantly elevated in patients (GD002, GD004-GD007) after ITNK infusion on day 3 based on Benjamini–Hochberg-adjusted *p* value. **C** Representative CT scans and PET-CT (MRI) of patients before (baseline) and after ITNK infusion at indicated time points post initial ITNK infusion. Tumors in patients GD004, GD005, GD006, GD007 and GD008 were stabilized (all tumors increased less than 20% in size), and GD002 achieved partial remission (PR), as revealed by both CT and PET-CT scan imaging diagnostic analysis at 16 months (34.6% decrease in tumor size), whereas tumors in patients GD001, GD003 and GD009 progressed following ITNK treatment (all tumors increased more than 30% in size). Red circles indicate the monitored tumor sites. **D** Percentages of ITNK cytotoxicity for patients with progressing diseases and patients with stable diseases. The assay was performed against K562 cells at an E: T ratio equal to 1:1 measured 24 h after coculture. Data are shown as the mean ± SD; *P* = 0.0082 (stable vs. progressing); unpaired t test, **P ≤ 0.01

The eight patients received intravenous or intra-arterial infusion of 0.31–102.04 × 10^6^ ITNKs per kg of body weight after cyclophosphamide (Cy) and/or fludarabine (Flu) lymphodepletion [86] (Table 1). In all cases, patients readily recovered from cell infusion-related side effects such as slight fever, fatigue, or muscle pain and were released after at least 1 week of inpatient observation (Table 1). Serum levels of 40 cytokines, including IL-6, a common indicator of immunotherapy response and cytokine release syndrome (CRS) [87], were measured in patients before and at different times after ITNK infusion (Fig. S11C and Table S9). Elevated levels of serum IL-6 were detected in seven of nine ITNK-treated patients (Fig. 6A). Of interest, the patients with the highest concentration of IL-6 (GD003) suffered only slight fever, fatigue and muscle pain for 2 days (Table 1 and Fig. 6A). Notably, several cytokines, including PDGF-BB, MIP-1β, GM-CSF, M-CSF and TNF-α, were significantly elevated in GD002 patients from day 3 post ITNK infusion onwards (Fig. 6B and S11C and Table S9), consistent with in vitro assays showing GM-CSF secretion (Fig. 4B).

No patients experienced CRS, neurotoxicity, or other severe adverse effects attributed to the cell infusion (Table 1, also described in the Materials and Methods section). They were subjected to clinical outcome evaluation according to the RECIST 1.1 standards at 1 month post infusion. Computed tomography (CT) scan imaging diagnostic analysis showed that tumors in patients GD004, GD005, GD006, GD007, and GD008 were clinically stable post ITNK treatment (Fig. 6C and S11D). Notably, patient GD002, who had undergone relapse with sacrum metastases after previous surgery, chemotherapy, and radiotherapy, achieved partial remission (PR) as revealed by both MRI and PET-CT scan imaging after 16 months. This subject had been intravenously infused 14 times with ITNKs but without any other medical treatment (Fig. 6C). In patient GD003, the tumor marker CA199 decreased in serum 1 month after receiving three doses of ITNKs by hepatic artery and ascending aortic injection (Fig. S11E). However, his tumors, as well as patient GD009, continued to progress (Fig. 6C and S11D). The overall characteristics of the patients and their clinical outcomes are summarized in Table 1. Retrospective analyses showed that stable responses are more likely to be achieved in patients whose ITNKs lysed K562 cells more efficiently and whose tumors exhibited high levels of NCR ligands and HLA-I expression (Table 1, Fig. 6D). In particular, the cytotoxicity of ITNKs in subject GD002 was the most potent and this patient achieved PR (Table 1). Taken together, the results of our preliminary clinical investigation indicated that ITNK infusions following Cy/Flu lymphodepletion are well tolerated and provided clinical benefit in 6/9 patients and one patient achieved partial remission.

## Discussion

Here, we demonstrate that acute loss of BCL11B reprograms mature human T cells into ITNKs, which express a diverse TCR repertoire and both T- and NK cell-associated genes and exhibit functional properties of both cell types (Table S11). Down-regulation of BCL11B also occurs naturally in the NKG2C^+^CD8^+^ T cells with an NK-like genetic signature and phenotype in HCMV-seropositive individuals [88]. The alterations in gene expression caused by BCL11B deletion in human T cells resemble, in various aspects, how Bcl11b ablation affects murine T cells [16–18], suggesting evolutionarily conservation. Innate lymphoid cells (ILCs) are a recently identified family of lymphoid effector cells comprising both “cytotoxic” ILCs (NK cells) and “helper” ILCs. The orchestration of ILC and CD4^+^ T cell subset differentiation is remarkably similar [89, 90]. Thus, similar to Th1 cells, ILC1 cells express TBX21 and produce IFN-γ [91], while ILC2 development requires Bcl11b [92]. ILC3 cells in turn express RORγt [93], which is absent from CD4^+^ ITNKs. However, future studies are required to compare the transcriptomes of CD4^+^ ITNKs with the different types of ILC cells.

Human ITNKs effectively eliminate leukemia and solid tumor-derived cells in culture. ITNKs expressed both TCRs and NCRs and efficiently killed leukemia cells in vitro through these TCRs and NCRs, such as NKp30 and NKp46 (Figs. 4A-D and S5D). Consistent with these findings, murine ITNKs use their TCR to recognize and lyse lymphoma cells expressing MHC-I molecules in vitro [16]. Human ITNKs were also found to target tumor cells in organoids and after transplantation into xenograft models. Of note, although ITNKs did not lyse K562 or primary HCC organoids as efficiently as NK cells did in vitro (Figs. 4C and S5E), they were superior in suppressing the growth of K562 tumors and multiple types of solid tumors in xenograft models (Figs. 4E-H, S5J). The differences between in vitro and in vivo antitumor effects may be due to the greater proliferation of ITNKs than NK cells in vivo. The mechanisms underlying ITNK activation and elimination of tumors in an in vivo microenvironment demand further investigation. ITNKs could be derived from various T cell subsets, including CD8, CD4, and γδT cells, and found to be functionally heterogeneous. CD8^+^ ITNKs expressed NKp46 and lysed K562 cells efficiently, while CD4^+^ ITNKs did not. However, CD4^+^ ITNKs secreted Th1 cytokines, including TNF-α (Fig. S5A, B). Further preclinical studies are required to identify the ITNK subsets with the most potent antitumor effects.

Our preliminary clinical results with infusions of ITNKs against solid tumors showed that they are safe and elicited clinical benefits in 6/9 patients. No clonal T/NK malignancies were observed in the limited number of phase I trial recipients treated to date, but long-term follow-up studies will be needed for all gene-modified cellular therapies to monitor possible late events. Optimization of CRISPR/Cas9 delivery into T cells, such as through the use of ribonucleoprotein (RNP) [9], may not only prevent plasmid-induced toxicity but also increase ITNK output by improving the deletion efficiency of BCL11B in T cells. With an improved ITNK manufacturing protocol, we can avoid undesired variability in dose levels and further escalate ITNK dosages, given that ITNKs were well tolerated in this study. In addition, due to the high lentiviral transduction efficiency of ITNKs, we can generate CAR-ITNKs to improve the specificity of ITNK recognition against tumors and introduce suicide genes such as inducible Caspase 9 (iCasp9) into ITNKs to avoid potential T cell leukemogenesis.

Human ITNKs express functional T cell receptors and NK cell receptors, and effectively kill both blood and solid cancer cells in culture, organoids, and mouse xenograft models. Remarkably, ITNKs resulted in tumor stabilization in six out of nine patients with advanced solid tumors in a preliminary clinical trial. Critically, no severe adverse effects have been observed in all these patients. ITNKs thus offers several major potential advantages over the immune cells currently being test clinically, e.g. CAR-T, CAR-NK: Robust proliferation, potent killing capacity, broad killing spectrum, no obvious severe adverse effects, and allowing further genetic engineering for example CAR-ITNKs to enhance specific killing. We think ITNK cells could serve as a more feasible and powerful choice compared to T cells or NK cells for clinicians to apply to general oncologic practice for multiple types of hematological or solid tumors, especially for those cancers with low scores of HLA-I or/and high scores of NCR ligands.

## Conclusions

Taken together, these studies show that ITNKs may provide a new cellular source for adoptive cell therapy.

## Supplementary Information

**Additional file 1: Table S1.** Downregulated differential expression genes (DEGs) in CD4 ITNKs compared to CD4 T cells, |Log2FC| > 1, padj< 0.05.

**Additional file 2: Table S2.** KEGG analysis of differential expression genes (DEGs) in CD8 ITNKs compared to CD8 T cells.

**Additional file 3: Table S3.** Upregulated differential expression genes (DEGs) in ITNKs compared to NK cells, |Log2FC| > 1, pval< 0.05.

**Additional file 4: Table S4.** Phenotyping characterization of sgRNA-ctrl and sgRNA-BCL11B edited T cells before in vivo infusion.

**Additional file 5: Table S5.** Whole genome sequencing analysis on predicted on- and off-target sites.

**Additional file 6: Table S6.** Characterizations of patients with autologous ITNK treatment.

**Additional file 7: Table S7.** ITNK cell product release specifications.

**Additional file 8: Table S8.** Characteristics of the infused ITNKs in the clinical trial.

**Additional file 9: Table S9.** Longitudinal measurement of relative levels for circulating cytokines/chemokines/growth factors in serum of patient GD001 before and after infusion.

**Additional file 10: Table S10.** Antibodies and sgRNAs used in this study.

**Additional file 11: Figure S1.** Inactivating *BCL11B* in human T cells by CRISPR/Cas9. **Figure S2.** ITNKs are derived from CD4^+^ and CD8^+^ T cells. **Figure S3**. ITNKs are derived from different T cell subsets. **Figure S4.** Immunophenotypic characteristics of ITNKs using CyTOF. **Figure S5.** Evaluating antitumor effects of ITNKs. **Figure S6.** Evaluating antitumor effects of CAR-ITNK cells. **Figure S7.** Evaluating kinetics and safety of clinical-grade ITNKs. **Figure S8.** Clinical trial protocol schematic. **Figure S9.** CONSORT statement/diagram. **Figure S10.** Immunohistochemistry staining of tumor tissues from patients. **Figure S11.** Clinical characteristics of patients treated with ITNK cells.

**Additional file 12: Table S11.** Biological characterizations of human ITNK, T, and NK cells.

## Data Availability

The sequencing datasets generated in this publication have been deposited in the NCBI Gene Expression Omnibus (GEO) or Sequence Read Archive (SRA) and are accessible under the following GEO series accession numbers: scRNA-seq: SRP293602., and WGS–seq: GSE143367. Bulk RNA-seq: HRA001256 data is publicly accessible at https://ngdc.cncb.ac.cn/gsa-human. Available upon request.

## Abbreviations

ITNK: Induced T-to-natural killer cells
ACT: Adoptive cell therapy
CAR: Chimeric antigen receptor
TCR: T cell receptor
FBS: Fetal bovine serum
GMP: Good Manufacturing Practice
NPC: Nasopharyngeal carcinoma
HCC: Hepatocellular carcinoma
CB: Cord blood
MAIT: Mucosal-associated invariant T
CyTOF: Cytometry by time of flight
PCA: Principal component analysis
DEGs: Differentially expressed genes
ADCC: Antibody-dependent cell-mediated cytotoxicity
PDOs: Patient-derived tumor organoids
scFv: Single chain fragment variation
BLI: Bioluminescence imaging
NSI: NOD/SCID/IL2RG^−/−^
GVHD: Graft-versus-host disease
LDLRs: Low-density lipid receptors
VSV-G: Vesicular stomatitis virus G protein
Cy: Cyclophosphamide
Flu: Fludarabine
CRS: Cytokine release syndrome
CT: Computed tomography
