# Supplementary material for: Human induced-T-to-natural killer cells have potent anti-tumour activities
Source: Biomark Res. 2022 Mar 24;10:13. doi: 10.1186/s40364-022-00358-4 (PMC8943975; doi:10.1186/s40364-022-00358-4)
Supplement: Supplementary file 4 — Additional file 4: Table S4. Phenotyping characterization of sgRNA-ctrl and sgRNA-BCL11B edited T cells before in vivo infusion. [file 40364_2022_358_MOESM4_ESM.docx]

**Table S4. Phenotyping characterization of sgRNA-ctrl and sgRNA-BCL11B edited T cells before in vivo infusion.**

| Phenotyping characterization | sgRNA-ctrl T | | sgRNA-BCL11B T | | P value | Significant |
| --- | --- | --- | --- | --- | --- | --- |
|  | means | s.d. | means | s.d. |  |  |
| CD3^+^NKp30^+^ | 12.70 | 5.55 | 54.17 | 16.77 | 0.0001 | *** |
| CD3^+^NKp30^-^ | 84.50 | 6.32 | 41.97 | 15.14 | <0.0001 | *** |
| CD3^+^CD4^+^NKp30^+^ | 11.23 | 7.78 | 37.67 | 15.75 | 0.0422 | * |
| CD3^+^CD8^+^NKp30^+^ | 84.43 | 8.62 | 51.67 | 15.16 | 0.0045 | ** |
| CD3^+^CD4^+^NKp30^-^ | 42.80 | 16.82 | 65.87 | 10.77 | 0.1207 | ns |
| CD3^+^CD8^+^NKp30^-^ | 54.10 | 17.10 | 30.67 | 10.61 | 0.1084 | ns |
| CD3^+^CD4^+^NKp30^+^CD45RO^+^CD45RA^-^ | 20.93 | 2.55 | 4.76 | 3.96 | 0.6008 | ns |
| CD3^+^CD4^+^NKp30^+^CD45RO^-^CD45RA^+^ | 45.07 | 1.10 | 67.10 | 16.45 | 0.1621 | ns |
| CD3^+^CD8^+^NKp30^+^ CD45RO^+^CD45RA^-^ | 1.73 | 1.57 | 1.67 | 1.78 | >0.9999 | ns |
| CD3^+^CD8^+^NKp30^+^ CD45RO^-^CD45RA^+^ | 87.10 | 5.21 | 78.33 | 5.91 | 0.9944 | ns |
| CD3^+^CD4^+^ NKp30^-^CD45RO^+^CD45RA^-^ | 54.33 | 4.77 | 23.03 | 15.35 | 0.0077 | ** |
| CD3^+^CD4^+^ NKp30^-^CD45RO^-^CD45RA^+^ | 21.83 | 4.35 | 39.63 | 17.72 | 0.4497 | ns |
| CD3^+^CD8^+^ NKp30^-^ CD45RO^+^CD45RA^-^ | 13.87 | 1.14 | 14.17 | 9.47 | >0.9999 | ns |
| CD3^+^CD8^+^ NKp30^-^ CD45RO^-^CD45RA^+^ | 66.20 | 2.57 | 46.60 | 4.04 | 0.3039 | ns |

Statistics of the percentages of CD3^+^NKp30^+^, CD3^+^NKp30^-^, CD4^+^, CD8^+^, T memory (CD45RO^+^CD45RA^-^) and T effector (CD45RO^-^CD45RA^+^) cells in sgRNA-ctrl and sgRNA-BCL11B edited T cells. *P<0.05, **P<0.01, ***P<0.001 and N.S. means not statistics difference.
