# Supplementary material for: Human induced-T-to-natural killer cells have potent anti-tumour activities
Source: Biomark Res. 2022 Mar 24;10:13. doi: 10.1186/s40364-022-00358-4 (PMC8943975; doi:10.1186/s40364-022-00358-4)
Supplement: Supplementary file 5 — Additional file 5: Table S5. Whole genome sequencing analysis on predicted on- and off-target sites. [file 40364_2022_358_MOESM5_ESM.docx]

**Table S5. Whole genome sequencing analysis on predicted on- and off-target sites**

| Sequence | Indels | Gene Name | Mutation  Type |
| --- | --- | --- | --- |
| ATGGTGGGTGGCCCCGAAGCAGTGTGGCGGCAGCTTGGGTGCCTGCTATGAC  --------------------80--------------------------------------------------CTTGGGTGCCTGCTATGAC  --------------------79-------------------------------------------------GCTTGGGTGCCTGCTATGAC | 2 | BCL11B | 79-80 bps  del |
| GGGACTCAGGGTGAGGGTCAGACGGAGGCTCCCTTTGGATGCCAGTGTCAGTT  GGGACTCAGGGTGAGGGTCAGACGGAG----5----CTTTGGATGCCAGTGTCAGTT  GGGACTCAGGGTGAGGGTCAGACGGAGGCTCC-2-TTGGATGCCAGTGTCAGTT | 2 | BCL11B | 2-5 bps  del |
| ATTTAAAAAAAATCAAAAGCAGTGTGGC--CAGCTTGG CCTTGATTGCCTAGG  ATTTAAAAAAAATCAAAAGCAGTGTGGC--CAGCTTGG CCTTGATTGCCTAGG | 0 | ABO | NA |
| CATCATCATCATAATCAAGCAGTGTGGCTGGCAGCTGGGGGCTAGCAGCCTAGT  CATCATCATCATAATCAAGCAGTGTGGCTGGCAGCTGGGGGCTAGCAGCCTAGT | 0 | MN1 | NA |
| TCTCCCCGCAGGTGTG--GCAGTGTGGCGGCAGCATGGAGGTGCTGCCCTGCT  TCTCCCCGCAGGTGTG--GCAGTGTGGCGGCAGCATGGAGGTGCTGCCCTGCT | 0 | GALNT9 | NA |
| AAAACTCAAAAGCCAG-AGCTGTGTGGCAGCAGCTTGGATGATAGAGGCTTTC AAAACTCAAAAGCCAG-AGCTGTGTGGCAGCAGCTTGGATGATAGAGGCTTTC | 0 | MAFTRR | NA |
| TGAACGTAGAAGCCCGAAGCATTGTGGAGG-AGCTAGGGCCTGATTTGAGAGG  TGAACGTAGAAGCCCGAAGCATTGTGGAGG-AGCTAGGGCCTGATTTGAGAGG | 0 | KCNQ3 | NA |
| CAAACCCCCTCTATTGAAGCAGTGTGGCGACAT-TGGGAAAAGGATGGTCTTT  CAAACCCCCTCTATTGAAGCAGTGTGGCGACAT-TGGGAAAAGGATGGTCTTT | 0 | GRIP1 | NA |
| AGGCTTCAAGGTCTGGAAGCAGG-TGGAGGCAGCTAGGAGTCTGACTACTGTG  CTGTTTTTAAGTCAGGACCTTGACCTGCGAACCTGGGGAGACAGGAAGAGCTT | 0 | LRRK2 | NA |
| GGACACAGATAAAGAGGTGAGA-GGAGGCTCCCTTAGGCTGCTAGGGAGGGGT  GGACACAGATAAAGAGGTGAGA-GGAGGCTCCCTTAGGCTGCTAGGGAGGGGT | 0 | MEGF10 | NA |
| TGACCTCAACCAGGCAGTCAGA-GGAGGCTCCCCTAGGAAACAAATGATGAT TGACCTCAACCAGGCAGTCAGA-GGAGGCTCCCCTAGGAAACAAATGATGAT | 0 | CACNA1D | NA |
| GAACGTTCTTTTAAGGTAGAGACGGAAGCTCCCTTGGGTGGAATAAGAGAATC  GAACGTTCTTTTAAGGTAGAGACGGAAGCTCCCTTGGGTGGAATAAGAGAATC | 0 | GRIK4 | NA |
| AGCTTGCAACAGAGTGGTCAGAACGGAGGGCCCCTTGGGCCCCCTTCCATTCAG  AGCTTGCAACAGAGTGGTCAGAACGGAGGGCCCCTTGGGCCCCCTTCCATTCAG | 0 | PTPRN2 | NA |
| CCAGCAGCTGGAAGAGGCAGGACGGAGCCTCCCTTGGGGCCTTCAGAGGGAAC  CCAGCAGCTGGAAGAGGCAGGACGGAGCCTCCCTTGGGGCCTTCAGAGGGAAC | 0 | SCMH1 | NA |
| CACTGTCTCCTTAGTGGGGAGGCTGAGGCTCCCTTGGGGTCTGCCCGCTGGGG  CACTGTCTCCTTAGTGGGGAGGCTGAGGCTCCCTTGGGGTCTGCCCGCTGGGG | 0 | PIGQ | NA |
| AGTGGGGGCTCCCTGGGGGAGTCGGGGGCTCCCTTGGGAAGCTGAGGGGTGGG  AGTGGGGGCTCCCTGGGGGAGTCGGGGGCTCCCTTGGGAAGCTGAGGGGTGGG | 0 | ACSS2 | NA |

T cells from Donor 1 and Donor 3 were transduced with sgRNA-*BCL11B* or sgRNA-control on day 1. Two batches of sorted ITNKs (NKp46^+^CD3^+^, purity>90%) from sgRNA-*BCL11B* edited T cells and two batches of purified T cells (CD3^+^) from sgRNA-control transduced T cells were subjected high-throughput genomewide sequencing at 30$\times$ coverage on day 14 to evaluate the off-target effects of gene editing. After removing low-quality reads we obtained averagely 111,287.73 Mb clean reads. The clean reads of sample had high Q20 (97.3%) and Q30 (89.5%), which showed high sequencing quality. A total of 16 predicted on- or off- target sites with indels detected within 15 bp were listed. The off-target sequence was in blue and the underlined bases represent PAM sequence of NGG. Mismatches to the gRNAs were highlighted in red. The indels detected by whole genome sequencing was shown in lower lines. NA: Not available.
