# Supplementary material for: Human induced-T-to-natural killer cells have potent anti-tumour activities
Source: Biomark Res. 2022 Mar 24;10:13. doi: 10.1186/s40364-022-00358-4 (PMC8943975; doi:10.1186/s40364-022-00358-4)
Supplement: Supplementary file 6 — Additional file 6: Table S6. Characterizations of patients with autologous ITNK treatment. [file 40364_2022_358_MOESM6_ESM.docx]

**Table S6. Characterizations of patients with autologous ITNK treatment**

| Patient ID | Body weight（kg） | Metastasis | Prior therapy/Surgery | Mode of administration | ITNK (%)^d^ | Infusion times |
| --- | --- | --- | --- | --- | --- | --- |
|  |  |  |  |  |  |  |
| GD001 | 72 | Lung, parotid | Local radiotherapy, surgical resection | I.V. | 40.7 | 1 |
|  |  |  |  |  |  | 2 |
|  |  |  |  |  |  | 3 |
| GD002 | 62 | Sacrum | mFOLFOX6 adjuvant chemotherapy, local radiotherapy | I.V. | 54.6 | 1 |
|  |  |  |  |  | 54.6 | 2 |
|  |  |  |  |  | 35.1 | 3 |
|  |  |  |  |  | 36.6 | 4 |
|  |  |  |  |  | 17.6 | 5 |
|  |  |  |  |  | 36.8 | 6 |
|  |  |  |  |  | 42.5 | 7 |
|  |  |  |  |  | 35.0 | 8 |
|  |  |  |  |  | 50.1 | 9 |
|  |  |  |  |  | 42.4 | 10 |
|  |  |  |  |  | 44.6 | 11 |
|  |  |  |  |  | 49.0 | 12 |
|  |  |  |  |  | 49.0 | 13 |
|  |  |  |  |  | 49.0 | 14 |
| GD003 | 48 | Liver, lung, bone, pancreas | Epirubicin, platinum, ifosfamide adjuvant chemotherapy, radiofrequency ablation, HIFU, resection, TOMO radiation | I.A. ^a^ | 10.1 | 1 |
|  |  |  |  |  | 11.9 | 2 |
|  |  |  |  |  | 42.6 | 3 |
| GD004 | 65 | Liver, bone | Albumin paclitaxel, bevacizumab, pembrolizumab, temozolomide, dacarbazine, cisplatin, vindesine, semustine, ipilimumab, nivolumab, local microwave ablation, radiation | I.V. | 20.7 | 1 |
| GD005 | 60 | Omentum, ovaries | Oxaliplatin, tegafur, gimeracil, oteracil, potassium adjuvant chemotherapy | I.V. | 18.6 | 1 |
| GD006 | 45 | Peritoneal | Oxaliplatin, 5-Fu, irinotecan, raltitrexed, xeloda, surgical resection | I.A. ^b^ | 49.1 | 1 |
|  |  |  |  | I.V. | 29.3 | 2 |
|  |  |  |  |  | 11.6 |  |
| GD007 | 60 | Pleura, bone, adrenals | Erlotinib, osmertinib, Pemetrexed, carboplatin, apatinib | I.V. | 44.2 | 1 |
| GD008 | 47 | Liver，abdominal lymph nodes | mFOLFOX6 plus bevacitumab chemotherapy, local radiotherapy , transartery chemoebolization | I.T. ^c^ | 39.4 | 1 |
|  |  |  |  | I.V. | 39.4 | 2 |
| GD009 | 67 | upper palate mucosa | Dacarbazine, cisplatin, bevacizumab,oxaliplatin, epirubicin, ipilimumab | I.V. | 31.8 | 1 |
|  |  |  |  |  | 37.5 | 2 |

I.V. intravenous; I.A.^a^, Hepatic arterial and ascending aortic injection; I.A.^b^ uterine artery injection; I.T.^c^ intra-tumour injected; ITNK (%)^d^: Percentage of ITNKs was calculated as (the percentage of CD3^+^NKp30^+^ cells of sgRNAs-*BCL11B* transduced T cells minus the percentage of CD3^+^NKp30^+^ cells of sgRNA-contorl transduced T cells); ITNK dosage ($\times$10^6^/kg) ^d^: Dosage of ITNK cell infusion was calculated as (the number of infused total cells)$\times$(the percentage of ITNKs) divided by the body weight of the patient.
