# Supplementary material for: Human induced-T-to-natural killer cells have potent anti-tumour activities
Source: Biomark Res. 2022 Mar 24;10:13. doi: 10.1186/s40364-022-00358-4 (PMC8943975; doi:10.1186/s40364-022-00358-4)
Supplement: Supplementary file 7 — Additional file 7: Table S7. ITNK cell product release specifications. [file 40364_2022_358_MOESM7_ESM.docx]

| **Assay** | **Release Specification** |
| --- | --- |
| Cell viability on sentinel vial | >70% |
| Transduction efficiency by FACS  (GFP expression 24h post electroporation) | >20% |
| Endotoxin | Negative |
| Mycoplasma | Negative |
| BACTEC culture | No growth at day 14 |
| IL-2 independent growth | No growth in absence of IL-2 at day 10 |
| Fungal culture | No growth at day 14 |
| % of ITNKs (CD3^+^NKp30^+^) | ≥10% |
| Disruption of *BCL11B* gene | Detectable disruption |
| In vitro killing against K562-GL | ≥40% at E: T ratio 1:1 |
| Residual sgRNA-*BCL11B* vector | < 50 average copies/ 1e6 cells |

**Table S7. ITNK cell product release specifications**
