# Supplementary material for: Human induced-T-to-natural killer cells have potent anti-tumour activities
Source: Biomark Res. 2022 Mar 24;10:13. doi: 10.1186/s40364-022-00358-4 (PMC8943975; doi:10.1186/s40364-022-00358-4)
Supplement: Supplementary file 10 — Additional file 10: Table S10. Antibodies and sgRNAs used in this study. [file 40364_2022_358_MOESM10_ESM.docx]

**Table S10. Antibodies and sgRNAs used in this study.**

| Targets | Clone | Tag | Vendor | Catolog Nmuber | Application |
| --- | --- | --- | --- | --- | --- |
| CD45 | HI30 | Y89 | Fluidigm | 3089003B | CyTOF |
| CD16 | 3G8 | 209Bi | Fluidigm | 3209002B | CyTOF |
| CD107a | H4A3 | 141Pr | Biolegend | 328635 | CyTOF |
| CD33 | WM53 | 174Yb | Biolegend | 303419 | CyTOF |
| CD69 | FN50 | 172Yb | Biolegend | 310939 | CyTOF |
| CD28 | CD28,2 | 147Sm | Biolegend | 302937 | CyTOF |
| OX40 | ACT35 | 170Er | Biolegend | 350015 | CyTOF |
| TIGIT | MBSA43 | 143Nd | eBioscience | 16-9500-82 | CyTOF |
| CCR7 | G043H7 | 171Yb | Biolegend | 353237 | CyTOF |
| NKp30 | 210845 | 156Gd | R&D | MAB1849-100 | CyTOF |
| NKp44 | 253415 | 164Dy | R&D | MAB22491 | CyTOF |
| NKp46 | 195314 | 153Eu | R&D | MAB1850-100 | CyTOF |
| NKG2D | 149810 | 152Sm | R&D | MAB139-100 | CyTOF |
| gdTCR | B1.1 | 106Cd | eBioscience | 14-9959-82 | CyTOF |
| CD45RA | HI100 | 110Cd | Biolegend | 304143 | CyTOF |
| CD45RO | UCHL1 | 111Cd | Biolegend | 304239 | CyTOF |
| CD3 | UCHT1 | 112Cd | Biolegend | 300443 | CyTOF |
| CD4 | RPA-T4 | 113Cd | Biolegend | 300541 | CyTOF |
| CD11b | ICRF44 | 114Cd | Biolegend | 301337 | CyTOF |
| CD15 | W6D3 | 116Cd | Biolegend | 323035 | CyTOF |
| CD62L | DREG-56 | 165Ho | Biolegend | 304835 | CyTOF |
| CD8 | SK1 | 162Dy | Biolegend | 344727 | CyTOF |
| CD19 | HIB19 | 142Nd | Biolegend | 302247 | CyTOF |
| CD11C | 3.9 | 146Nd | Biolegend | 301639 | CyTOF |
| CD25 | AF-223 | 169Tm | R&D | AF-223-NA | CyTOF |
| CD27 | O323 | 158Gd | Biolegend | 302839 | CyTOF |
| CD31 | WM59 | 145Nd | Biolegend | 303127 | CyTOF |
| CD34 | 581 | 148Nd | Biolegend | 343531 | CyTOF |
| CD38 | HIT2 | 167Er | Biolegend | 303535 | CyTOF |
| CD39 | A1 | 160Gd | Biolegend | 328221 | CyTOF |
| CD56 | HCD56 | 149Sm | Biolegend | 318345 | CyTOF |
| CD123 | 6H6 | 151Eu | Biolegend | 306027 | CyTOF |
| CD127 | A019D5 | 176Yb | Biolegend | 351337 | CyTOF |
| PD-1 | EH12.2H7 | 155Gd | Biolegend | 329941 | CyTOF |
| CTLA4 | AF386 | 161Dy | R&D | AF86-PB | CyTOF |
| TIM | F38-2E2 | 154Sm | Biolegend | 345019 | CyTOF |
| ICOS | 669222 | 168Er | R&D | MAB6975 | CyTOF |
| 4-1BB | AF838 | 173Yb | R&D | AF838 | CyTOF |
| GITR | 10416 | 159Tb | R&D | MAB689 | CyTOF |
| CD7 | CD7-6B7 | 166Er | Biolegend | 343111 | CyTOF |
| CD3 | OKT3 | PE-Cy7 | Biolegend | 317334 | FACS |
| CD4 | OKT4 | PerCP-cy5 | Biolegend | 317428 | FACS |
| CD8 | RPA-T8 | FITC | Biolegend | 301006 | FACS |
| CD8 | BC96 | PE | Biolegend | 302606 | FACS |
| CD56 | 5.1H11 | PE | Biolegend | 306722 | FACS |
| Nkp30 | P30-15 | PE | Biolegend | 325208 | FACS |
| Nkp30 | P30-15 | APC | Biolegend | 325210 | FACS |
| Nkp44 | P44-8 | PE | Biolegend | 325110 | FACS |
| Nkp44 | P44-8 | APC | Biolegend | 325108 | FACS |
| Nkp46 | 9E2 | PE | Biolegend | 331908 | FACS |
| Nkp46 | 9E2 | APC | Biolegend | 331918 | FACS |
| TCR γ/δ | B1 | PE | Biolegend | 331209 | FACS |
| IFNɣ | B27 | PE | Biolegend | 506507 | FACS |
| CD107a | H4A3 | PerCP-Cy7 | Biolegend | 328616 | FACS |
| CD45RO | UCHL1 | BV421 | Biolegend | 304223 | FACS |
| CD45RA | HI100 | APC-CY7 | Biolegend | 304127 | FACS |
| anti-NKp30 | MAB1849-SP |  | R&D | MAB1849 | Function |
| anti-NKp46 |  |  | Miltenyi Biotec | 130-094-483 | Function |
| anti-CD3 | UCHT1 |  | Biolegend | 300401 | Function |
| anti-CD28 | C28.2 |  | Biolegend | 302901 | Function |
| Anti-HER2 |  |  | Selleck | A2007 | Function |
| Anti-NKp30 | P30-15 |  | Biolegend | 325223 | Blocking |
| Anti-NKp46 | 9E2 |  | Biolegend | 331947 | Blocking |
| mIgG1 | MOPC-21 |  | Biolegend | 400101 | Control |
| GAPDH | mAbcam9484 |  | Abcam | ab9484 | Western blot |
| BCL11B | 25B6 |  | Abcam | ab18465 | Western blot |
| sg-*BCL11B* | GAAGCAGTGTGGCGGCAGCT | | GGTCAGACGGAGGCTCCCTT | | Function |
| sg-ctrl | CCGGGTCTTCGAGAAGACCT | |  | | Function |
