# Supplementary material for: Human induced-T-to-natural killer cells have potent anti-tumour activities
Source: Biomark Res. 2022 Mar 24;10:13. doi: 10.1186/s40364-022-00358-4 (PMC8943975; doi:10.1186/s40364-022-00358-4)

Supplementary figure 1

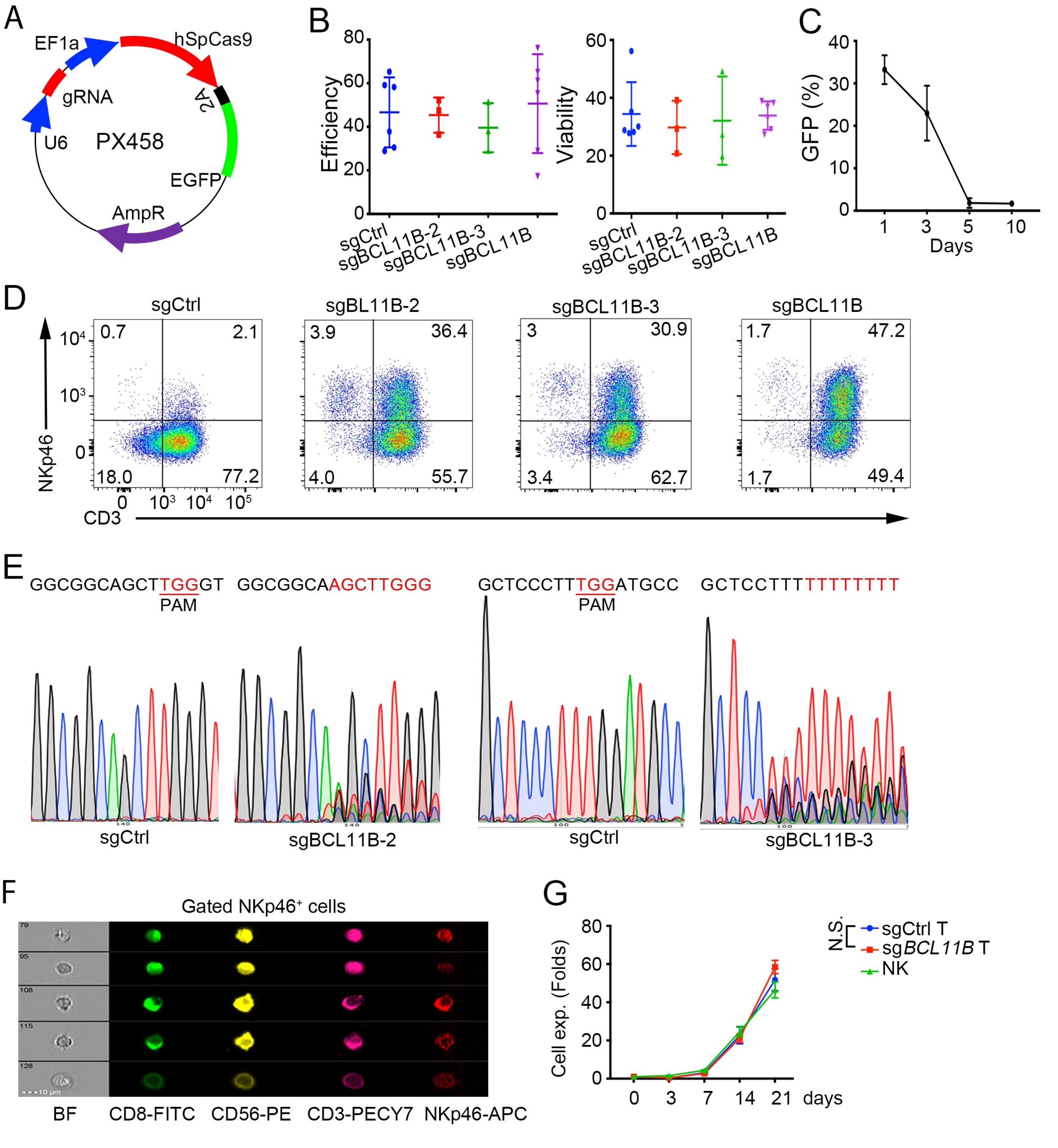

Supplementary figure 2

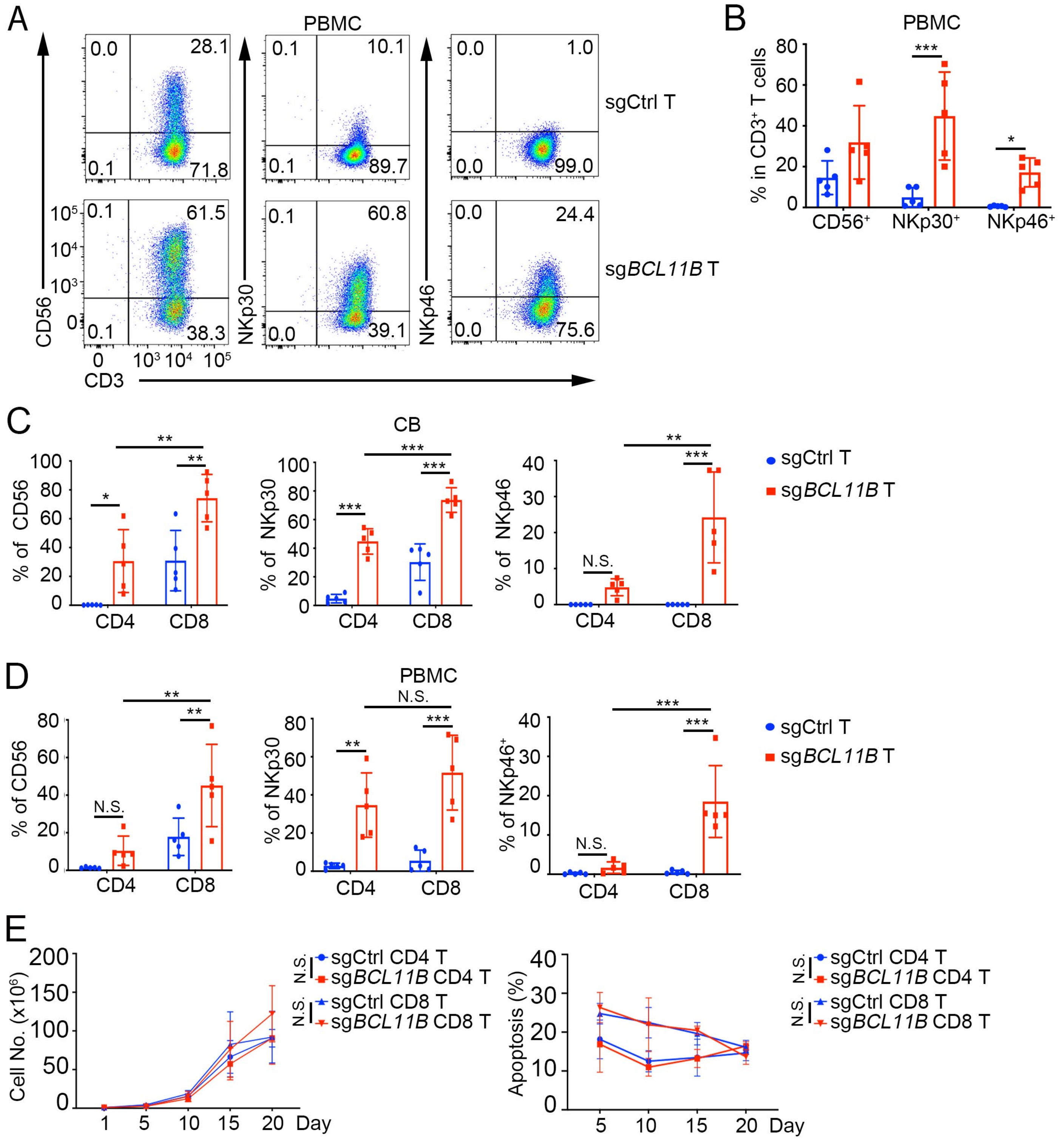

Supplementary figure 3

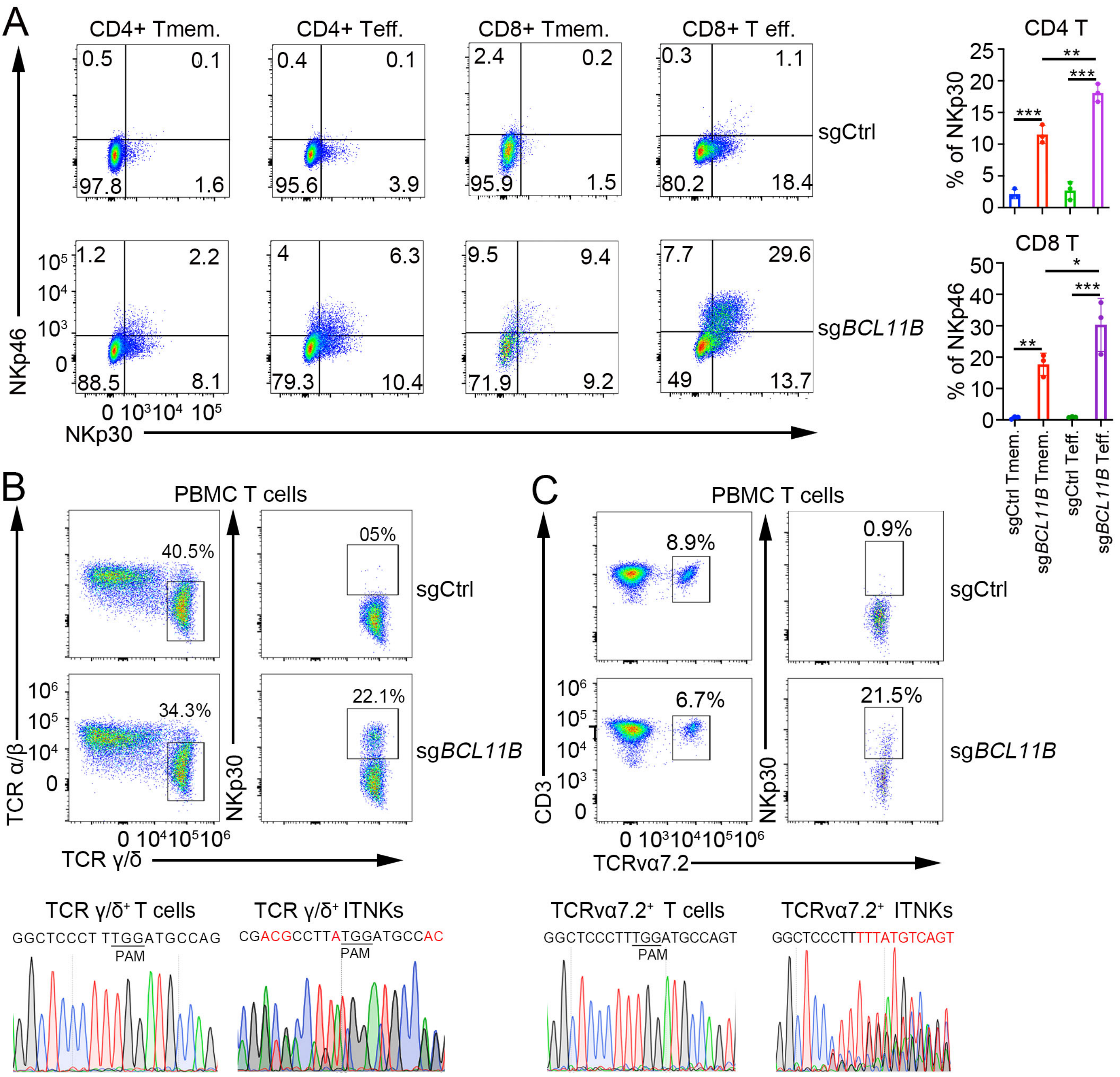

Supplementary figure 4

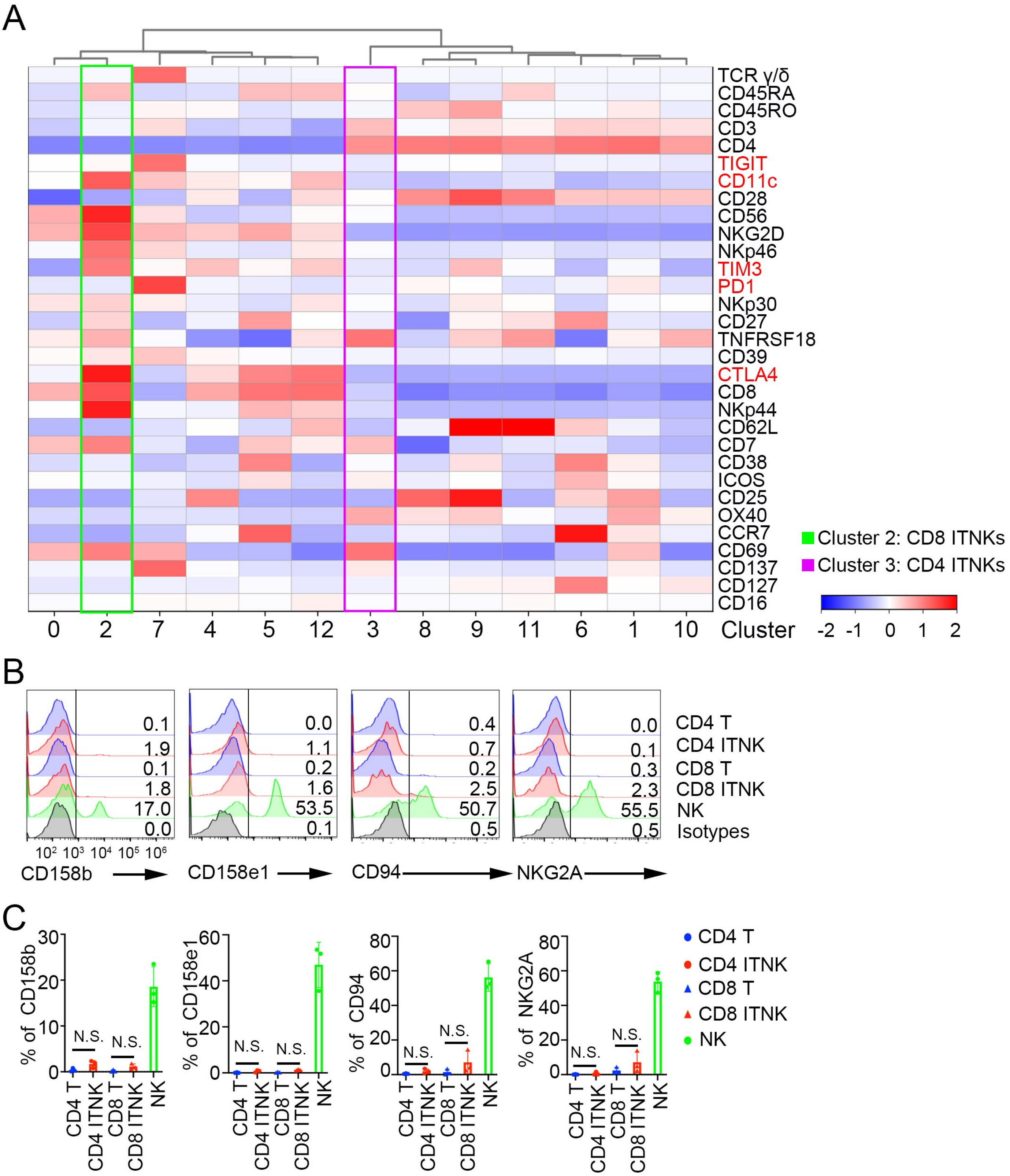

Supplementary figure 5

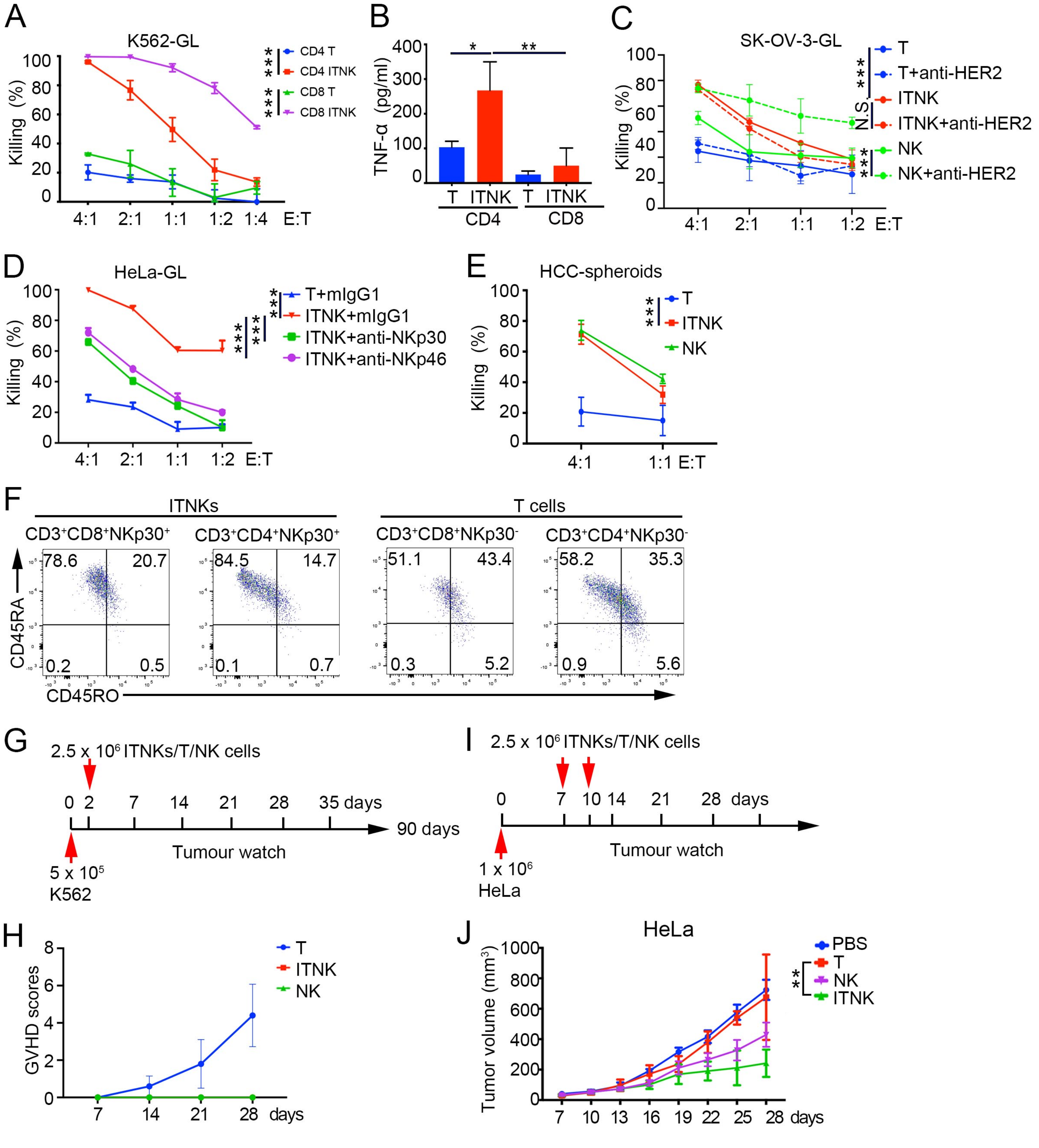

Supplementary figure 6

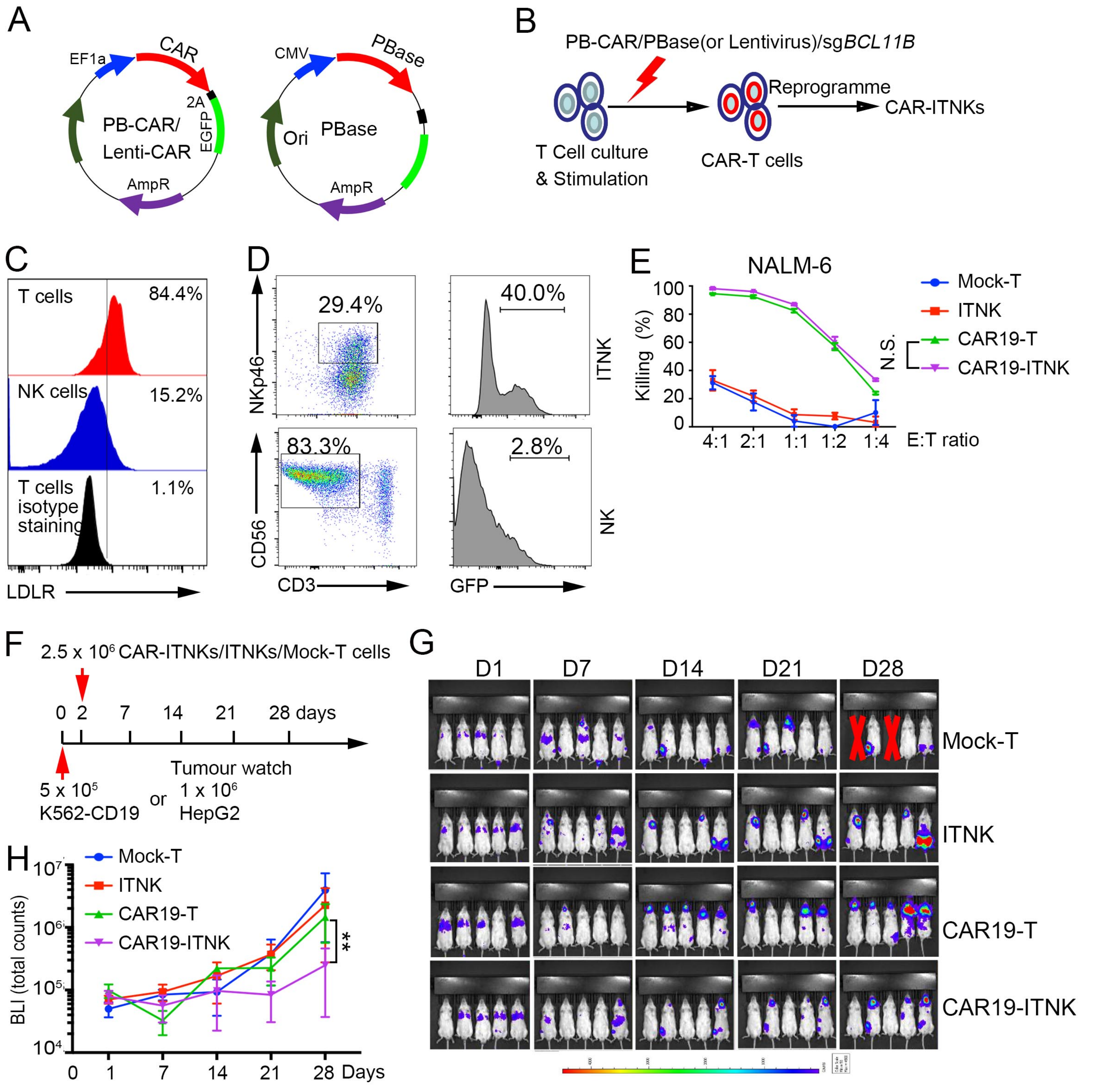

Supplementary figure 7

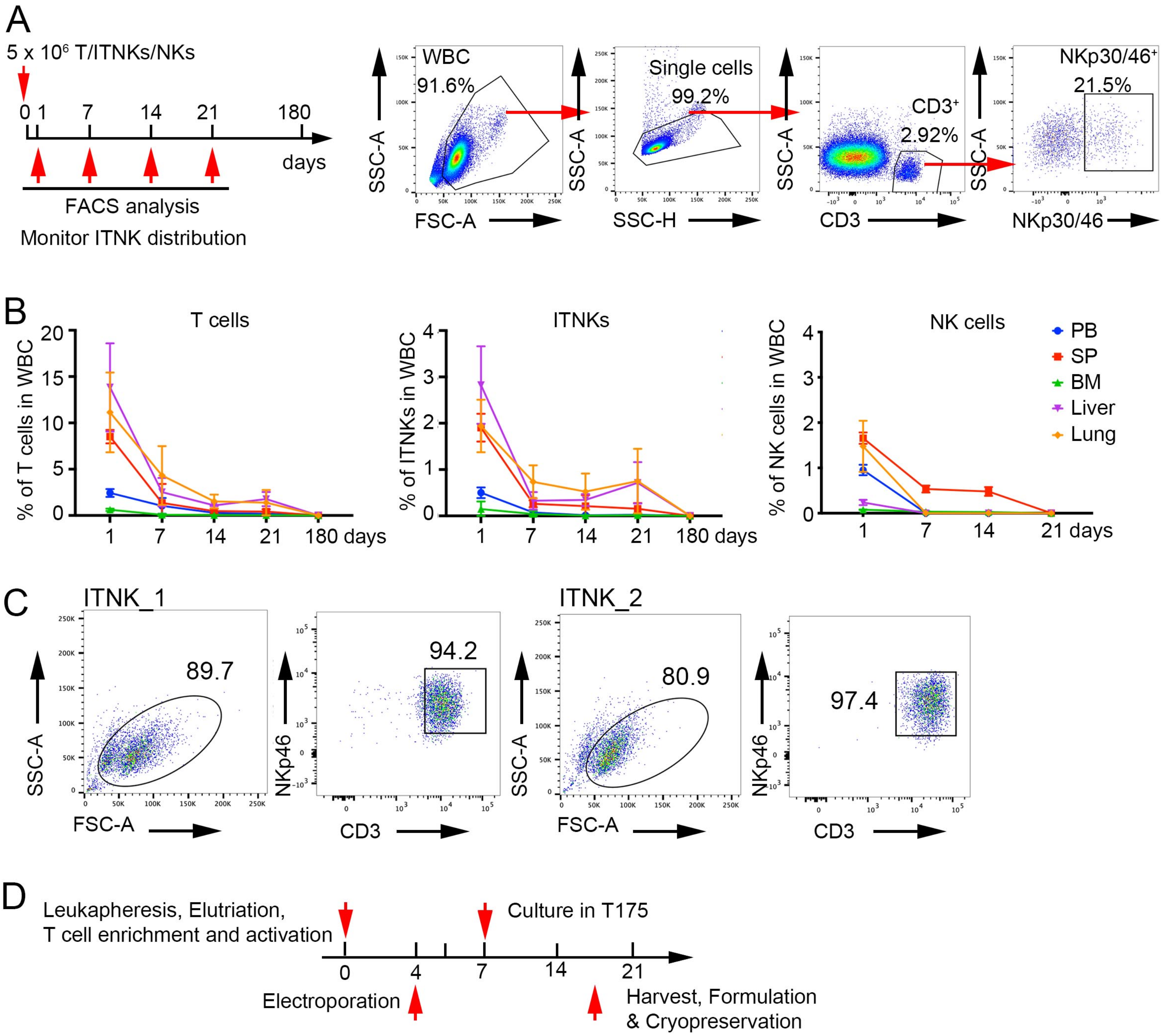

Supplementary figure 8

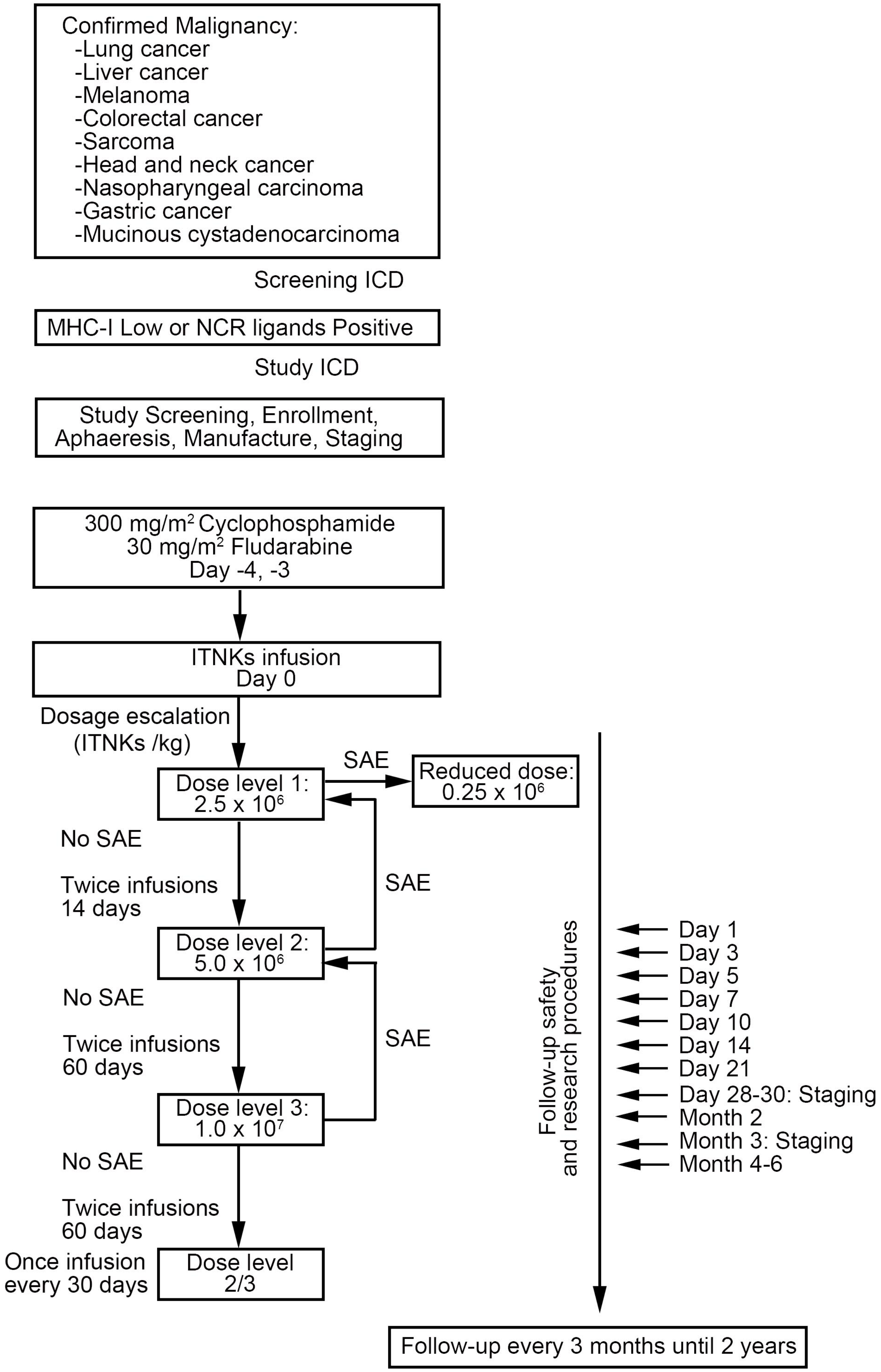

Supplementary figure 9

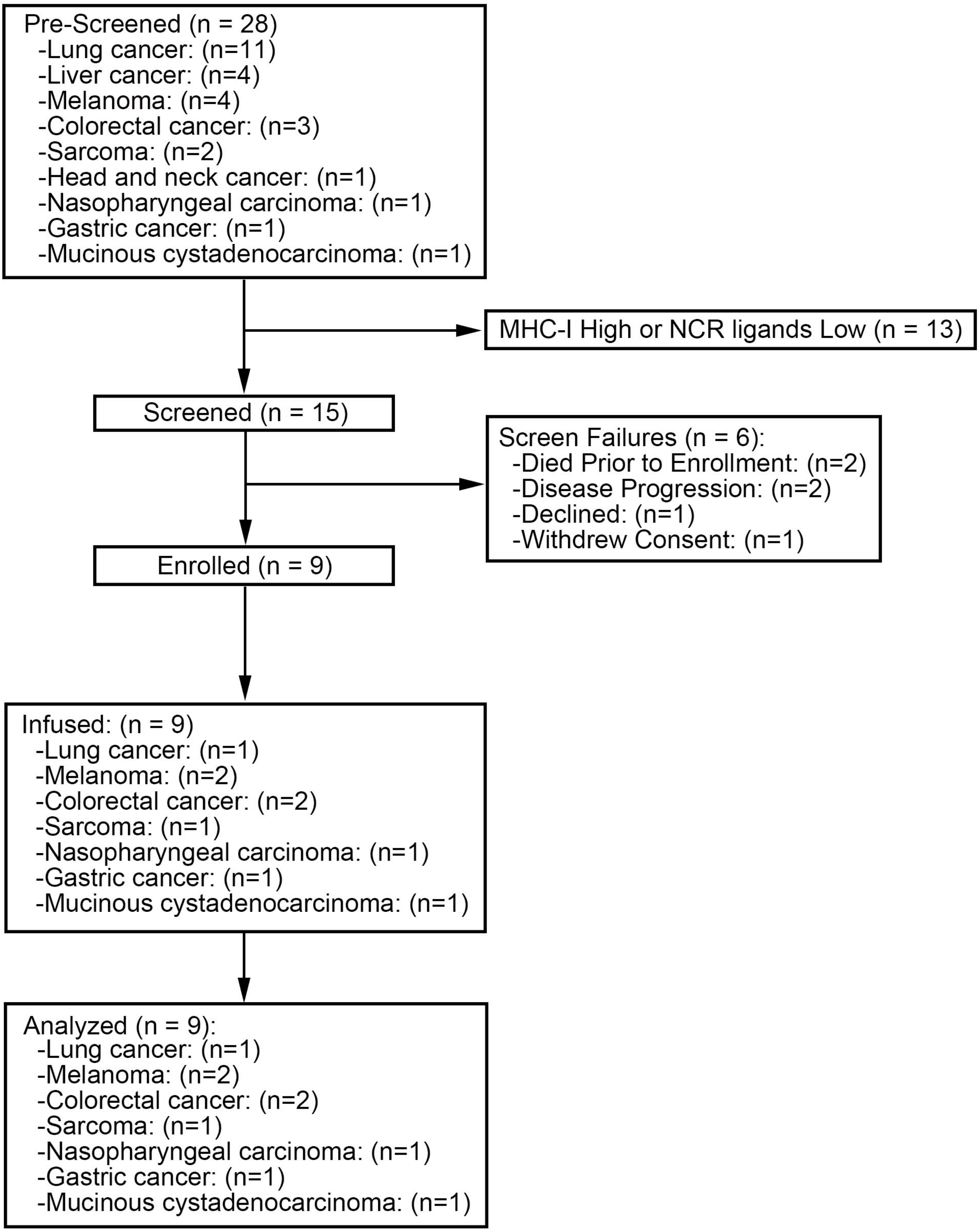

Supplementary figure 10

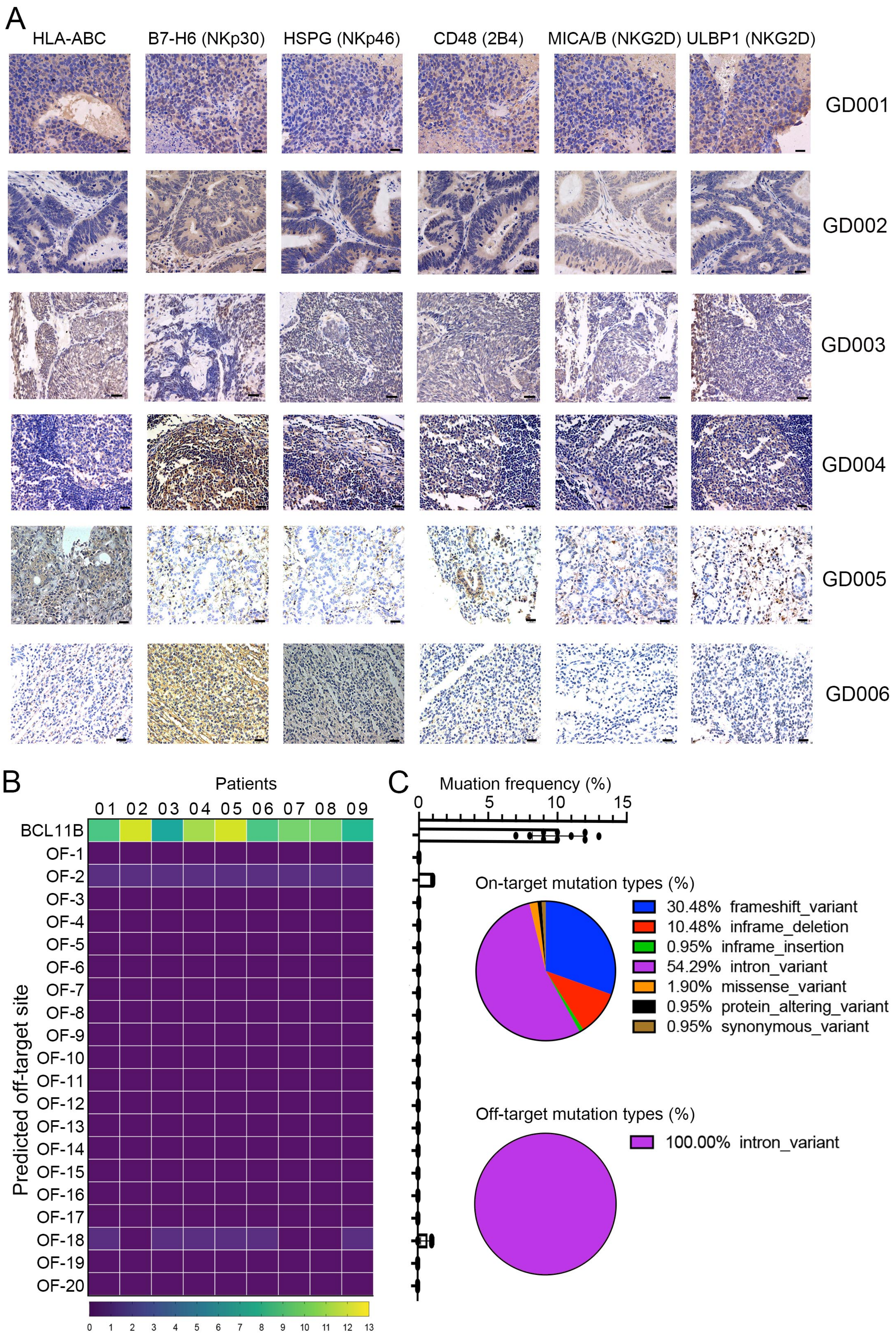

Supplementary figure 11

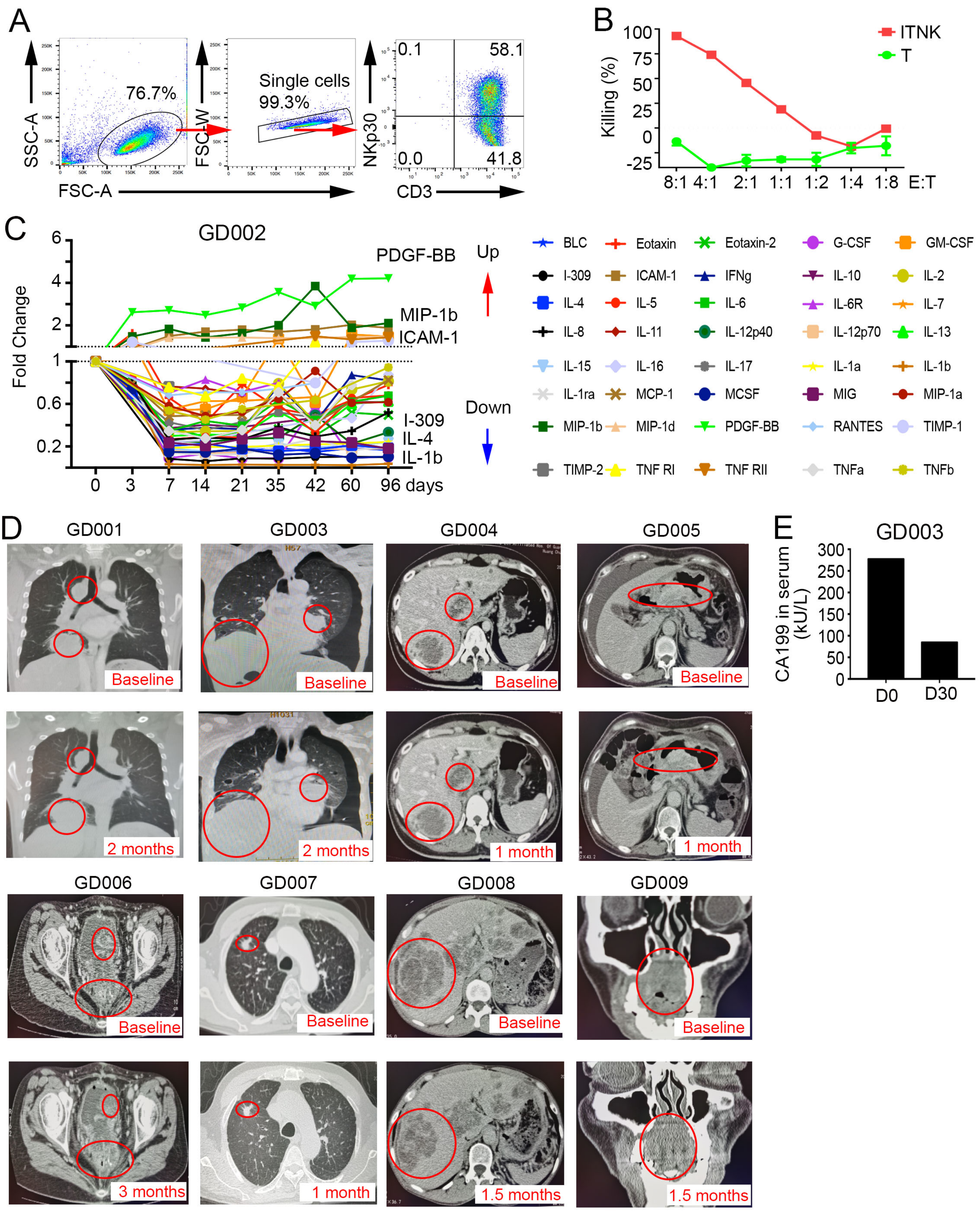

Supplement: Supplementary file 11 — Additional file 11: Figure S1. Inactivating BCL11B in human T cells by CRISPR/Cas9. Figure S2. ITNKs are derived from CD4+ and CD8+ T cells. Figure S3. ITNKs are derived from different T cell subsets. Figure S4. Immunophenotypic characteristics of ITNKs using CyTOF. Figure S5. Evaluating antitumor effects of ITNKs. Figure S6. Evaluating antitumor effects of CAR-ITNK cells. Figure S7. Evaluating kinetics and safety of clinical-grade ITNKs. Figure S8. Clinical trial protocol schematic. Figure S9. CONSORT statement/diagram. Figure S10. Immunohistochemistry staining of tumor tissues from patients. Figure S11. Clinical characteristics of patients treated with ITNK cells. [file 40364_2022_358_MOESM11_ESM.pdf]
