# Supplementary material for: Human induced-T-to-natural killer cells have potent anti-tumour activities
Source: Biomark Res. 2022 Mar 24;10:13. doi: 10.1186/s40364-022-00358-4 (PMC8943975; doi:10.1186/s40364-022-00358-4)
Supplement: Supplementary file 12 — Additional file 12: Table S11. Biological characterizations of human ITNK, T, and NK cells. [file 40364_2022_358_MOESM12_ESM.docx]

**Table S11. Biological characterizations of human ITNK, T, and NK cells**

|  | **ITNK** | **T** | **NK** |
| --- | --- | --- | --- |
| **Morphology** | Large granular lymphocytes | Small or non-granular lymphocytes | Large granular lymphocytes |
| **Surface**  **makers** | CD3^+^CD8^+^NKp46^+^/ CD3^+^CD4^+^NKp30^+^ | CD3^+^CD4^+^/ CD8^+^ | CD3^-^CD56^+^ |
| **TCR**  **repertoire** | Highly variable | Highly variable | - |
| **Activating**  **receptors** | NKp30/46, TCR | TCR | NKp30/44/46, NKG2C/E, NKG2D |
| **Inhibiting**  **receptors** | CTLA4, TIM3 | PD-1, CTLA4, LAG3, TIM3 | KIR, SIGLEC7/9,  CD94/NKG2A, |
| **Target recognition** | TCR-MHC, Activating KIR/ NCR | TCR-MHC | Activating KIR/ NCR |
| **Restriction**  **reactivity** | MHC independent | MHC positive | MHC negative |
| **Transcription**  **factors** | ID2, NFIL3, TCF1^low^ | GATA3,  BCL11B | ID2, NFIL3 |
| **Allogeneic effects** | Reduced | Yes | Reduced |
| **Functions** | Anti-tumour | Anti-tumour,  destroy infected cells, regulate immune responese | Anti-tumour,  destroy infected cells |

ITNK: Induced-T-to-Natural Killer Cells, TCR: T-cell receptor, MHC: major histocompatibility complex, KIR: killer-cell immunoglobulin-like receptors, NCR: natural cytotoxicity receptors
